# Drug Repurposing to Treat Glucocorticoid Resistance in Asthma

**DOI:** 10.3390/jpm11030175

**Published:** 2021-03-03

**Authors:** Alberta L. Wang, Ronald Panganiban, Weiliang Qiu, Alvin T. Kho, Geoffrey Chupp, Deborah A. Meyers, Eugene R. Bleecker, Scott T. Weiss, Quan Lu, Kelan G. Tantisira

**Affiliations:** 1 Channing Division of Network Medicine, Brigham and Women’s Hospital, Boston, MA 02115, USA; weiliang.qiu@gmail.com (W.Q.); alvin_kho@hms.harvard.edu (A.T.K.); scott.weiss@channing.harvard.edu (S.T.W.); 2 Division of Allergy and Clinical Immunology, Brigham and Women’s Hospital, Boston, MA 02115, USA; 3 Program in Molecular and Integrative Physiological Sciences, Departments of Environmental Health and Molecular Metabolism, Harvard T.H. Chan School of Public Health, Boston, MA 02115, USA; rpangan@hsph.harvard.edu (R.P.); qlu@hsph.harvard.edu (Q.L.); 4 Computational Health Informatics Program, Boston Children’s Hospital, Boston, MA 02115, USA; 5 Division of Pulmonary, Critical Care, and Sleep Medicine, Yale University School of Medicine, New Haven, CT 06520, USA; geoffrey.chupp@yale.edu; 6 Division of Genetics, Genomics and Precision Medicine, Department of Medicine, University of Arizona, Tucson, AZ 85724, USA; dameyers@email.arizona.edu (D.A.M.); erbleecker@email.arizona.edu (E.R.B.); 7 Division of Respiratory Medicine, Department of Pediatrics, University of California San Diego, San Diego, CA 92123, USA; ktantisira@health.ucsd.edu

**Keywords:** asthma, anti-asthmatic agents, drug repositioning, glucocorticoid effect

## Abstract

Corticosteroid resistance causes significant morbidity in asthma, and drug repurposing may identify timely and cost-effective adjunctive treatments for corticosteroid resistance. In 95 subjects from the Childhood Asthma Management Program (CAMP) and 19 subjects from the Severe Asthma Research Program (SARP), corticosteroid response was measured by the change in percent predicted forced expiratory volume in one second (FEV_1_). In each cohort, differential gene expression analysis was performed comparing poor (resistant) responders, defined as those with zero to negative change in FEV_1_, to good responders, followed by Connectivity Map (CMap) analysis to identify inversely associated (i.e., negatively connected) drugs that reversed the gene expression profile of poor responders to resemble that of good responders. Mean connectivity scores weighted by sample size were calculated. The top five drug compound candidates underwent in vitro validation in NF-κB-based luciferase reporter A549 cells stimulated by IL-1β ± dexamethasone. In CAMP and SARP, 134 and 178 respective genes were differentially expressed in poor responders. CMap analysis identified 46 compounds in common across both cohorts with connectivity scores < −50. γ-linolenic acid, ampicillin, exemestane, brinzolamide, and INCA-6 were selected for functional validation. γ-linolenic acid, brinzolamide, and INCA-6 significantly reduced IL-1β induced luciferase activity and potentiated the anti-inflammatory effect of dexamethasone in A549/NF-κB-luc reporter cells. These results demonstrate how existing drugs, including γ-linolenic acid, brinzolamide, and INCA-6, may be repurposed to improve corticosteroid response in asthmatics.

## 1. Introduction

Asthma is one of the most common chronic respiratory diseases worldwide affecting more than 339 million children and adults [1]. Inhaled corticosteroids (ICS) are considered one of the primary treatments for the control of asthma. However, up to 30–50% of asthmatics have poor response to corticosteroid treatment [2,3]. Corticosteroid refractory asthma represents about 50% of total costs for treating asthma [4]. While the new next generation biologics may help to address the issue of corticosteroid resistance, they remain very expensive and must be administered parenterally. Therefore, interrogations to identify alternatives for the treatment of steroid-refractory asthma are warranted. Drug repurposing studies in asthma to date have focused on identifying new drugs for the primary treatment of asthma and not for the treatment of resistance against corticosteroids or other classes of medications [5,6,7,8,9]. Gene expression studies have demonstrated an important role for transcriptomics in determining ICS treatment response, and drugs that reverse the gene expression profile of poor ICS responders to be more similar to that of ICS good responders may potentially restore treatment response [10,11,12].

The development of a novel drugs is expensive, time consuming, and associated with low success rates. On average, it takes 13–15 years and US $2–3 billion to get a new drug to market, and only 15% of drugs successfully progress to FDA approval [13,14]. Drug repositioning or repurposing reduces drug development time and costs 6.5 years and US $300 million by finding new indications for the 3000 existing approved drugs [13,14]. In fact, two-thirds of the applications approved by the FDA are new indications for existing drugs [15].

The Connectivity Map (CMap) at the Broad Institute is a genome-scale library of more than 1.5 million transcriptional responses to genetic and pharmacologic perturbations in cultured human cell lines that have been used to identify drug repurposing candidates in many fields and diseases, including psychiatry, cancer, diabetes, Alzheimer’s disease, and asthma [7,16,17,18,19,20,21,22,23]. The CMap dataset contains perturbational gene expression profiles from treatment of 54 cultivated cell lines with 5000 small-molecule compounds [16,17]. These reference gene expression profiles can be compared to gene expression profiles from a biological state of interest (i.e., poor corticosteroid response). Compounds that elicit a similar expression signature to the disease state of interest are termed positively connected, and compounds that elicit an expression signature inverse to the disease state of interest are termed negatively connected [16,17]. To date, CMap has predominantly been used to identify novel repurposed drugs for the primary treatment of disease, rather than focusing on compounds that may be used to overcome drug resistance to improve existing treatments for a disease. In this study, we used CMap to identify negatively connected drug compounds that reversed the gene expression signature of corticosteroid poor responders to resemble that of corticosteroid good responders. The top identified drug compounds were validated in in vitro cell line experiments providing novel proof-of-concept of repurposing existing compounds to alleviate corticosteroid resistance in asthmatics.

## 2. Materials and Methods

### 2.1. Subjects

The Childhood Asthma Management Program (CAMP) and Severe Asthma Research Program (SARP) III comprise the two study cohorts. CAMP was a multicenter randomized control trial of inhaled budesonide, nedocromil, or placebo for mild-to-moderate persistent asthma in North America [24]. Immortalized B cells were cultured from 145 subjects randomized to inhaled budesonide, and the subjects were divided into tertiles based on ICS response measured by the change in percent predicted forced expiratory volume in one second (FEV_1_) from baseline at enrollment to month two on inhaled budesonide (Figure S1a) [25]. Parental informed consent with subject assent was obtained, and the study was approved by each CAMP study center institutional review board (IRB) and by the CAMP Coordinating Center (Mass General Brigham IRB 1999P0015492). The 48 children in the lowest tertile of ICS response were considered poor ICS responders (median change in FEV_1_ % predicted of −2.7 (±6.7), and the 47 children in the highest tertile were considered good responders (median change in FEV_1_ % predicted of 15.8 (±16.7), Kruskal–Wallis Test *p*-value < 0.001). For both poor and good responders, the immortalized B cells for each subject were split into two equal halves. One-half was treated with dexamethasone, and the other half was treated with sham. After 6 h, expression levels of 22,184 gene probes were measured using the Illumina HumanRef8 v2 BeadChip (San Diego, CA, USA). Each pair of dexamethasone-treated and sham-treated arrays was in the same batch. Details of the gene expression data quality control have been published [25]. The data were variance-stabilized then quantile normalized and log2-transformed, and 20,917 gene probes passed quality control.

SARP is a multisite longitudinal observational cohort study of children and adults that investigates the mechanisms of severe asthma [26]. From the Wake Forest University site, data were available on 19 adult subjects who underwent extensive phenotyping at baseline and at two research visits prior to and two weeks after an intramuscular injection of 40mg triamcinolone acetonide [27]. Sputum induction was performed before and after the corticosteroid evoked phenotype, and RNA sequencing was obtained on the sputum cells (Appendix A). The IRB at each center approved the study (Wake Forest University IRB 00021507). The data were normalized using the DESeq2 R package then log_2_-transformed, and 26,422 transcripts present at both visits passed quality control [27,28]. The 19 subjects were divided into good and poor responders based on the change in percent predicted FEV_1_ before and after steroid administration, which was the same measurement used in CAMP. Good responders (*n* = 8) had a change in percent predicted FEV_1_ greater than zero (median 2.6 (±3.9)), and poor responders (*n* = 11) had a change in percent predicted FEV_1_ less than or equal to zero (median −4.0 (±5.6), Kruskal–Wallis Test *p*-value < 0.001) (Figure S1b).

### 2.2. Statistical Analysis

All statistical analyses were performed in R (Vienna, Austria). Univariate T-tests, Wilcoxon rank-sum tests, χ^2^ tests, and Fisher’s exact tests were used to evaluate the demographics of good and poor responders within CAMP and SARP. Differential gene expression analysis was performed separately for CAMP and SARP to detect genes differentially expressed between poor and good responders. The outcome variable was the difference in gene expression pre- and post-corticosteroid administration. Multivariable linear regression controlling for age, sex, and ancestry was performed using the limma and iCheck R packages [29,30]. Due to small sample sizes, no genes were differentially expressed after false discovery rate adjustment for multiple testing, and genes with a nominal *p*-value < 0.01 were taken forward to CMap analysis. The differentially expressed genes were categorized into two groups, genes over-expressed in poor responders and genes under-expressed in poor responders, for input into CMap. The org.Hs.eg.db R package was used to annotate Entrez gene IDs to HGNC gene symbols and chromosome locations [31].

### 2.3. Connectivity Map Analysis

CMap analysis was performed using CLUE (CMap and LINCS Unified Environment), a cloud computing platform at the Broad Institute that facilitates access and manipulation of CMap data [32]. CMap was queried separately using the gene expression profiles of corticosteroid poor responders from CAMP and SARP to identify similar and dissimilar reference perturbagen signatures. CMap input requires Entrez gene IDs, and therefore, differentially expressed gene without Entrez IDs cannot be queried. Out of all available cell lines in CMap, the A549 cell line was selected for analysis because it is a lung-derived airway epithelial cell, and asthma is, at least in part, a disease of the airway epithelium. Furthermore, the anti-inflammatory effects of inhaled corticosteroid treatment are largely targeted at the airway epithelium, and for consistency, A549 cells were used for functional validation studies as described below. The CMap query outputs a connectivity score for each drug compound perturbagen, ranging from −100 to +100. A negative connectivity score of −100 indicates that the compound completely reversed the gene expression profile of poor responders to resemble that of good responders by inducing the expression of genes that are down-regulated in poor responders and suppressing the expression of genes that are up-regulated in poor responders. Hence, drug compounds with negative connectivity scores less than −50 were prioritized as these drugs had the highest likelihood of reversing corticosteroid resistance. Next, weighted mean connectivity scores were calculated based on sample size for the drug compounds with connectivity scores less than −50 in both cohorts.

### 2.4. In Vitro Validation

The top 10 available drug compounds with the highest weighted mean negative connectivity scores were screened, and five were chosen for in vitro validation based on safety data in humans, mechanistic plausibility, and drug availability. Functionally validation was performed using a previously described glucocorticoid-mediated tethered transrepression of NF-κB activity assay [33]. A549/NF-κB-luc reporter cells were treated with the selected compounds at indicated concentrations for 1 h. Standard drug concentrations were used (1 µM or 1µg/mL) and increased up to the limit where no cell death occurred. These A549/NF-κB-luc reporter cells are responsive to interleukin (IL)-1β stimulation, which is reduced by dexamethasone, a glucocorticoid receptor agonist, indicating that these cells exhibit glucocorticoid-mediated tethered transrepression of NF-κB [33]. One hour post-treatment, the cells were stimulated with 5 ng/mL IL-1β ± 2 nM dexamethasone. Luciferase assays were performed after 18 hours of treatment using the Luciferase Assay System (Promega, Madison, WI, USA), and luciferase activity was measured using the Synergy 2 plate reader (BioTek, Winooski, VT, USA). The luciferase activity in untreated (no drug compounds) cells exposed to 5 ng/mL IL-1β was normalized to 1. The luciferase activities in cells treated with the drug compounds were compared to that of untreated cells. Data were expressed as means ± standard errors. Statistical significance was evaluated using Student’s *t*-test (*n* = 4, *p* < 0.05).

## 3. Results

### 3.1. Demographics

The characteristics of the CAMP and SARP subjects included in this study are presented in Table 1. CAMP was composed of children (mean age 8.7 (±2.2) years) with mostly moderate persistent asthma. The subjects from SARP were all adults (mean age 46.5 (±8.4) years) with mostly severe persistent asthma. Both cohorts had a female sex and European ancestry predominance. In CAMP, the pretreatment percent predicted FEV_1_ and FEV_1_ to forced vital capacity ratio were lower in good responders compared to poor responders, and overall, lung function measurements were preserved in CAMP. The demographics of SARP did not vary across responder status. To mitigate the heterogeneity across populations, differential gene expression and CMap analyses were performed independently in each cohort, and then, the independent sets of drug compounds identified on CMap analysis were consolidated.

### 3.2. Differential Gene Expression Analysis

Differential gene expression analysis in CAMP revealed 72 under-expressed and 62 over-expressed genes in corticosteroid poor responders compared to good responders (Table S1). In SARP, 66 genes were under-expressed and 112 genes were over-expressed in corticosteroid poor responders compared to good responders (Table S2).

### 3.3. Connectivity Map Analysis

Next, the differentially expressed genes in CAMP and SARP were separately analyzed in CMap. Out of 2395 perturbagen compounds in the A549 cell line in CMap, 673 compounds had a negative connectivity score in CAMP, of which 277 compounds had a connectivity score less than −50 (Table S3). In SARP, 684 compounds had a negative connectivity score, and 261 compounds had a connectivity score less than −50 (Table S4). CAMP and SARP had 200 compounds in common with connectivity scores less than zero and 46 compounds in common with connectivity scores less than −50 (Table 2). Among the identified candidate compounds, eight were glucocorticoid receptor agonists, which is consistent with the direction of effect expected and a logical verification of our results. The compounds were prioritized based on weighted mean negative connectivity scores, and out of the top 10 drug compounds, γ-linolenic acid, ampicillin, exemestane, brinzolamide, and INCA-6 were selected for functional validation based on safety data in humans, mechanistic plausibility, and drug availability. Enrofloxacin, the compound with the highest weighted mean negative connectivity score, was not selected because it is a veterinary antibiotic. Due to limited data in humans and their conflicting mechanisms, serotonin receptor antagonist SB-203186 and serotonin receptor agonist dipropyl-5ct were not selected for functional validation.

### 3.4. In Vitro Validation

**Figure 1:**
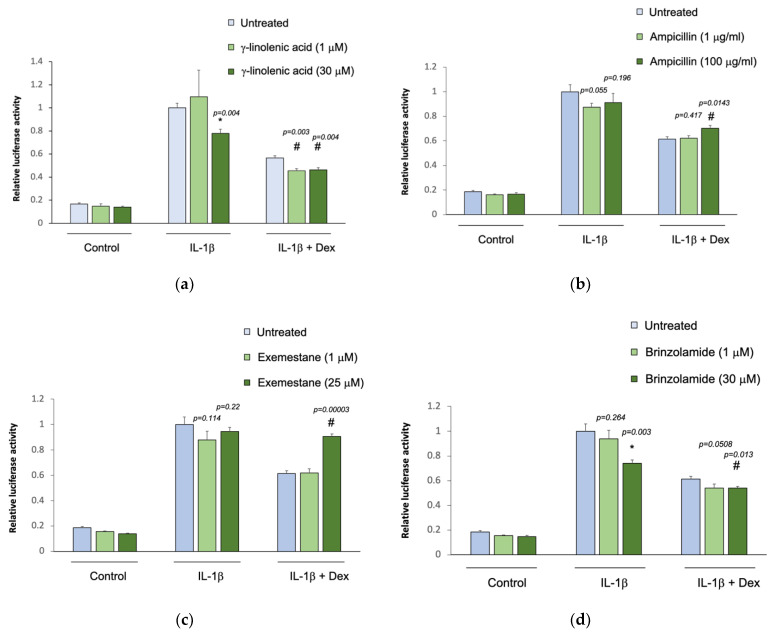

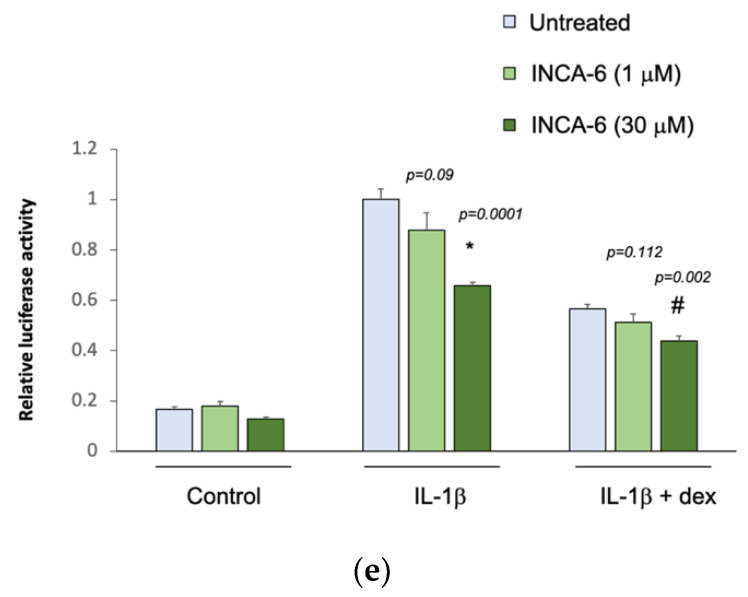
Effect of the selected drug compounds on the response of NF-κB reporter cells to IL-1β ± dexamethasone (dex). In vitro validation of the top five drug compounds from the Connectivity Map analysis in the Childhood Asthma Management Program and Severe Asthma Research Program III: (**a**) γ-linolenic-acid, (**b**) ampicillin, (**c**) exemestane, (**d**) brinzolamide, and (**e**) INCA-6. The luciferase activities of reporter cells treated with the drug compounds were compared to that of untreated cells. Data represent means ± standard errors compared using *t*-tests. * *p* < 0.05 (drug compounds vs. untreated in cells exposed to IL-1β alone); ^#^ *p* < 0.05 (drug compounds vs. untreated in cells exposed to IL-1β ± dex).

The five selected drug compounds were tested for their ability to modulate the anti-inflammatory effect of glucocorticoids using an assay based on the tethered transrepression of NF-κB activity by dexamethasone [33]. γ-linolenic acid, brinzolamide, and INCA-6 significantly reduced IL-1β induced luciferase activity in A549/NF-κB-luc reporter cells (Figure 1). Treatment with 30 μM of γ-linolenic acid led to a 20% relative reduction in luciferase activity after IL-1β stimulation compared to untreated cells (*p* = 0.004), and in the presence of dexamethasone, γ-linolenic acid treated cells had a significantly greater absolute reduction in luciferase activity (*p* = 0.004). Treatment with 30 μM of brinzolamide led to a 25% relative reduction in luciferase activity after IL-1β stimulation compared to untreated cells (*p* = 0.003), and the addition of dexamethasone led to a significantly greater absolute reduction in luciferase activity in brinzolamide treated cells (*p* = 0.013). Treatment with 30 μM of INCA-6 led to a 35% relative reduction in luciferase activity after IL-1β stimulation compared to untreated cells (*p* = 0.0001), and the addition of dexamethasone led to significantly greater absolute reduction in luciferase activity in INCA-6 treated cells (*p* = 0.002). Ampicillin and exemestane did not change IL-1β induced luciferase activity, and in the presence of dexamethasone, individual treatment with the highest concentrations of ampicillin and exemestane increased IL-1β induced luciferase activity compared to untreated samples (*p* = 0.0143 and *p* = 0.00003, respectively). Together, these data suggest that γ-linolenic acid, brinzolamide, and INCA-6 can each act as independent anti-inflammatory agents and synergistically augment the anti-inflammatory effects of corticosteroids.

## 4. Discussion

Poor response to corticosteroid treatment causes significant morbidity and mortality in asthma, and no treatments exist to directly alleviate corticosteroid resistance [34]. Few drug repurposing studies have been performed in asthma, and none have studied the repurposing of existing drugs to alleviate corticosteroid resistance [5,6,7,8,9]. This study is unique in its investigation of drugs that can be used to improve existing asthma therapies. Most prior studies used a drug-based approach by selecting specific compounds, including antithyroid and antihelminth agents, to repurpose as a new indication for the treatment of asthma, whereas this study used a disease-based drug repurposing approach to investigate the novel indication of glucocorticoid resistance in asthma [5,6,7,8,9].

In asthmatics treated with corticosteroids, we determined the intraindividual gene expression changes differentiating poor from good corticosteroid response as measured by FEV_1_ and identified 200 existing compounds that reversed the gene expression profile of poor responders to resemble that of good responders. These identified drug compounds have the potential to be repurposed as adjunctive treatments to improve corticosteroid response. Moreover, three of the top five candidate compounds were validated in in vitro human airway epithelial cells. γ-linolenic acid, brinzolamide, and INCA-6 each possessed anti-inflammatory activity independent of dexamethasone administration and further reduced IL-1β induced NF-κB activation in the presence dexamethasone.

γ-linolenic acid is an omega-6 fatty acid with anti-inflammatory properties found in plant-based oils such as borage oil, blackcurrant seed oil, and evening primrose oil [35,36]. As part of the polyunsaturated fatty acid biosynthesis pathway, γ-linolenic acid is elongated to form dihomo-γ-linolenic acid, which in turn is metabolized into anti-inflammatory series 1 prostaglandins, in particular prostaglandin E_1_ (PGE_1_), and 15-hydroxyeicosatrienoic acid (15-HETrE) by cyclooxygenase-1 and -2 and 15-lipoxygenase, respectively [35,36,37]. Paradoxically, dihomo-γ-linolenic acid can also undergo desaturation to form arachidonic acid, a precursor of many pro-inflammatory lipid mediators [36,37]. However, γ-linolenic acid is predominantly anti-inflammatory as both PGE_1_ and 15-HETrE metabolites inhibit the biosynthesis of arachidonic acid-derived eicosanoids [37]. In addition, dietary supplementation with γ-linolenic acid in asthmatics has been shown to reduce inflammation by attenuating leukotriene production and improve both quality of life and symptom control, and in agreement with these prior studies our results demonstrate the ability of γ-linolenic acid to potentiate the anti-inflammatory effect of glucocorticoids in asthma [38,39,40,41].

Brinzolamide is a carbonic anhydrase inhibitor used to treat elevated intraocular pressure in ocular hypertension and glaucoma. Carbonic anhydrase catalyzes the conversion of carbon dioxide and water to carbonic acid and also plays an important role in regulating type 2 inflammation. Carbonic anhydrase expression is induced by type 2 cytokines and elevated in allergen-induced pulmonary eosinophils [42]. Moreover, carbonic anhydrase positively regulates mast cell development, and inhibition of carbonic anhydrase selectively reduces human mast cell development and allergen-induced mast cell responses [43]. In addition, the inhibition of carbonic anhydrase by acetazolamide has been demonstrated to protect against bronchoconstriction induced by sodium metabisulfite, cold air, and deep inspiration in asthma [44,45,46]. These anti-inflammatory and protective effects of carbonic anhydrase inhibitors in asthma support our findings of its ability improve glucocorticoid response.

INCA-6 competitively binds to and inhibits calcineurin to prevent the transcriptional activation of cytokines, including IL-2, IL-4, IL-5, and granulocyte-macrophage colony-stimulating factor, leading to a reduction in both T lymphocyte proliferation and eosinophil proliferation and survival [47]. Calcineurin inhibitors also inhibit the release of histamine and the generation of arachadonic-acid derived eicosanoids from mast cells and basophils [48]. Resistance to glucocorticoids is a class effect caused by reduced inhibition of T lymphocyte proliferation by glucocorticoids and is not due to altered drug pharmacokinetics or metabolism [49]. Treatment with anti-T-lymphocyte agents including the calcineurin inhibitor cyclosporine has been shown to inhibit T cell proliferation in both glucocorticoid sensitive and resistance asthmatics [49]. Cyclosporine has also been found to improve lung function and reduce chronic oral corticosteroid dose and exacerbation rate [50,51]. However, the routine use of calcineurin inhibitors such as cyclosporine and tacrolimus in the treatment of asthma has been limited by adverse effects such as renal impairment, hypertension, hypertrichosis, headache, and peripheral neuropathy [50,51]. Calcineurin inhibitors are favorable candidates for drug repurposing to improve glucocorticoid sensitivity, but the selection of patients and optimal dosage needs to be carefully determined.

Included among the top 46 compounds identified on CMap analysis (Table 2) were both serotonin receptor agonists and antagonists, calcium channel activators and blockers, and adrenergic receptor agonists and antagonists. These antagonistic results suggest that serotonin, calcium, and adrenergic signaling have an important yet complex role in regulating corticosteroid response in asthmatics. Further research is needed to confirm the anti-inflammatory effects of these additionally identified drug classes. Contrary to expectations based on CMap, ampicillin and exemestane at the highest concentrations increased luciferase activity in A549/NF-κB-luc reporter cells in in vitro experiments. Ampicillin is a semisynthetic antibiotic derived from penicillin, and its routine use to treat asthma exacerbations has not been shown to accelerate improvement [52]. Exemestane is an aromatase inhibitor that blocks the conversion of androgens to estrogen. Estrogen promotes type 2 airway inflammation in asthma, which supports a plausible anti-inflammatory role for exemestane [53]. These pro-inflammatory effects of ampicillin and exemestane were concentration-dependent, and a different effect may have been detected at lower concentrations that were not tested. In addition, in vitro cell lines may not replicate true biological conditions and a different effect of these drugs may also be found in vivo. Further in vivo experiments are needed to determine the safety, efficacy, and optimal dosing of the identified compounds as adjunctive therapies to improve glucocorticoid response in asthma.

Genetic mutations affecting glucocorticoid resistance are only found in a small minority of asthmatics and do not fully explain the steroid resistance phenotype seen in asthma. Therefore, we focused on gene expression changes, which capture a wide variety of upstream processes, including environmental, genetics, and epigenetic causes that affect corticosteroid response. In addition, by examining relative instead of absolute corticosteroid resistance, our results have greater applicability across the spectrum of corticosteroid resistance in asthmatics. Strengths of this study include the inclusion of diverse cohorts, allowing for broad generalizability of our results across age, sex, ancestry, and asthma severity. Because of the heterogeneity between CAMP and SARP, differential gene expression and CMap analyses were performed separately in each cohort instead of a replication analysis, which produced two independent sets of negatively connected drug compounds. On direct comparison of these two independent sets of negatively connected drug compounds, 200 identical drug compounds were identified indicating the potential for these drugs to reverse poor corticosteroid response in asthmatics across a spectrum of age ranges, asthma severity, cell types, gene expression platforms, and corticosteroid response measurements. Furthermore, both the CMap analysis and in vitro validation were performed in A549 cells, which are representative of type II alveolar epithelial cells and suitable for studying drug delivery and metabolism in the pulmonary epithelium [54].

Important limitations to this study exist. CMap contains more than 2000 small-molecule compound perturbational signatures in A549 cells, and drug compounds not in the database were not investigated. CAMP used genotyping microarray data, and SARP used RNA sequencing data. It is possible that the use of RNA sequencing in both populations in addition to larger sample sizes would have increased the discovery of genes associated with steroid treatment response and hence, CMap hits. In addition, we only considered FEV_1_ response as an index of steroid nonresponsiveness. Previous work from our group has shown that steroid nonresponsiveness can present as other phenotypes not evaluated here [55]. Finally, although a glucocorticoid-resistant in vitro model was not used, the three compounds (γ-linolenic acid, brinzolamide, and INCA-6) that synergistically augmented the anti-inflammatory effect of dexamethasone also demonstrated anti-inflammatory activity at high concentrations independent of glucocorticoid administration and response. None of these theoretical limitations invalidate our overall approach and study design.

## 5. Conclusions

In summary, we identified several existing drugs with anti-inflammatory properties that may potentially alleviate glucocorticoid resistance in asthma. Experimental validation confirmed our results by demonstrating the potentiation of the effect of dexamethasone in pulmonary epithelial cells by γ-linolenic acid, brinzolamide, and INCA-6. Future in vivo studies are needed before these drugs can be approved as adjunctive therapies for asthma. Drug repurposing is timely and cost effective and has tremendous promise in treating poor glucocorticoid response. This novel approach can be generalized to treat resistance to standard therapies in diseases beyond asthma.

## Figures and Tables

**Table 1:** Demographics of Childhood Asthma Management Program (CAMP) and Severe Asthma Research Program (SARP).

	CAMP			SARP		
	Good Responders (*n* = 47)	Poor Responders (*n* = 48)	*p*-Value	Good Responders (*n* = 8)	Poor Responders (*n* = 11)	*p*-Value
**Age** (mean ± SD)	9.0 (±2.2)	8.5 (±2.1)	0.3	50.9 (±9.1)	43.3 (±6.6)	0.05
**Sex**			0.61			1.00
Female	25 (53.2%)	23 (47.9%)		7 (87.5%)	9 (81.8%)	
Male	22 (46.8%)	25 (52.1%)		1 (12.5%)	2 (18.2%)	
**Ancestry**			0.6			1.00
European	39 (83.0%)	42 (87.5%)		5 (62.5%)	8 (72.7%)	
African	5 (10.6%)	4 (8.3%)		3 (37.5%)	3 (27.3%)	
Hispanic	1 (2.1%)	2 (4.2%)		-	-	
Other	2 (4.3%)	0 (0.0%)		-	-	
**Asthma severity**			0.61			0.10
Mild	23 (48.9%)	21 (43.8%)		3 (37.5%)	1 (9.1%)	
Moderate	24 (51.1%)	27 (56.2%)		2 (25.0%)	1 (9.1%)	
Severe	-	-		3 (37.5%)	9 (81.8%)	
**Body mass index** (mean ± SD)	18.1 (±2.8)	18.0 (±2.9)	0.9	34.9 (±8.2)	35.3 (±8.3)	0.90
**Baseline serum IgE level** (kU/L, median ± IQR)	230.0 (±310.0)	150.0 (±236.0)	0.3	205.0 (±396.0)	36.0 (±195.0)	0.09
**Baseline serum eosinophil count** (cells/µL, median ± IQR)	493.0 (±481.0)	390.0 (±400.0)	0.1	244.0 (±220.0)	172.0 (±77.0)	0.08
**Baseline PC_20_** (median ± IQR)	0.6 (±1.0)	1.1 (±1.7)	0.05	0.4 (±0.0)	1.5 (±0.8)	0.50
**Pre-treatment FEV_1_ % predicted** (mean ± SD)	86.0% (±15.2%)	99.8% (±14.1%)	<0.001	72.5% (±22.5%)	82.7% (±11.9%)	0.20
**Post-treatment FEV_1_ % predicted** (mean ± SD)	101.0% (±11.5%)	100.5% (±15.1%)	0.8	76.5% (±23.6%)	73.6% (±17.9%)	0.80
**Pre-treatment FEV_1_/FVC** (mean ± SD)	75.1% (±9.9%)	80.8% (±7.4%)	0.002	70.4% (±11.0%)	75.8% (±8.6%)	0.20
**Post-treatment FEV_1_/FVC** (mean ± SD)	82.0% (±6.5%)	81.5% (±8.3%)	0.7	71.6% (±9.2%)	73.0% (±9.2%)	0.80

**Table 2:** Mean negative connectivity scores in CAMP and SARP weighted by sample sizes.

Name	Description	CAMP (*n* = 95) Connectivity Score	SARP (*n* = 19) Connectivity Score	Weighted Mean Connectivity Score
enrofloxacin	Bacterial DNA gyrase inhibitor	−96.51	−99.79	−97.06
SB-203186	Serotonin receptor antagonist	−93.70	−91.12	−93.27
γ-linolenic-acid	Anti-inflammatory omega-6 fatty acid	−94.78	−80.70	−92.43
dipropyl-5ct (3-(N,N-Dipropylaminoethyl)-1H-indole-5-carboxamide maleate)	Serotonin receptor agonist	−92.65	−83.03	−91.05
ampicillin	Bacterial cell wall synthesis inhibitor	−97.60	−56.59	−90.76
exemestane	Aromatase inhibitor	−86.59	−98.24	−88.53
brinzolamide	Carbonic anhydrase inhibitor	−86.81	−89.74	−87.30
INCA-6	Calcineurin inhibitor	−87.45	−78.14	−85.90
SCH-23390	Dopamine receptor antagonist	−88.17	−72.69	−85.59
brazilin	Nitric oxide production inhibitor	−86.00	−80.72	−85.12
pyrazinamide	Fatty acid synthase inhibitor	−82.85	−90.52	−84.13
vinburnine	Adrenergic receptor antagonist	−79.35	−99.25	−82.67
NS-1619	Calcium channel activator	−79.38	−98.59	−82.58
caffeine	Adenosine receptor antagonist	−78.83	−81.33	−79.25
masitinib	KIT inhibitor	−79.32	−76.71	−78.88
isoflupredone	Glucocorticoid receptor agonist	−82.06	−62.39	−78.78
fluocinonide	Glucocorticoid receptor agonist	−80.62	−66.33	−78.24
fluoxetine	Selective serotonin reuptake inhibitor	−74.30	−96.36	−77.98
quinpirole	Dopamine receptor agonist	−75.20	−90.40	−77.73
saquinavir	HIV protease inhibitor	−72.31	−94.39	−75.99
retinol	Retinoid receptor ligand	−72.33	−80.84	−73.75
doxapram	Potassium channel blocker	−74.67	−61.12	−72.41
somatostatin	Somatostatin receptor agonist	−68.97	−81.93	−71.13
thalidomide	TNF production inhibitor	−73.74	−57.45	−71.02
L-690488	Inositol monophosphatase inhibitor	−65.92	−86.80	−69.40
fludroxycortide	Glucocorticoid receptor agonist	−69.60	−58.68	−67.78
dexamethasone	Glucocorticoid receptor agonist	−63.63	−86.38	−67.42
nitrendipine	Calcium channel blocker	−70.41	−52.10	−67.36
acyclovir	DNA polymerase inhibitor	−64.90	−71.25	−65.96
DMAB-anabaseine	Adrenergic receptor agonist	−68.48	−51.34	−65.62
m-chlorophenylbiguanide	Serotonin receptor agonist	−61.73	−82.67	−65.22
SB-590885	RAF inhibitor	−65.27	−59.71	−64.34
trimebutine	Opioid receptor agonist	−58.98	−84.10	−63.17
teicoplanin	Bacterial cell wall synthesis inhibitor	−64.10	−56.12	−62.77
PD-102807	Acetylcholine receptor antagonist	−61.37	−61.17	−61.34
benzatropine	Acetylcholine receptor antagonist	−55.99	−81.83	−60.30
GBR-13069	Dopamine uptake inhibitor	−57.34	−70.82	−59.59
vinblastine	Microtubule inhibitor	−55.40	−76.83	−58.97
sulindac	Cyclooxygenase inhibitor	−58.98	−55.76	−58.44
dexketoprofen	Cyclooxygenase inhibitor	−59.67	−50.92	−58.21
hydrocortisone	Glucocorticoid receptor agonist	−50.67	−91.73	−57.51
fludrocortisone	Glucocorticoid receptor agonist	−50.42	−92.94	−57.51
OMDM-2	FAAH inhibitor	−57.04	−58.07	−57.21
diflorasone	Corticosteroid agonist	−54.44	−70.40	−57.10
beclometasone	Glucocorticoid receptor agonist	−52.13	−72.93	−55.60
marbofloxacin	Bacterial DNA gyrase inhibitor	−55.04	−51.70	−54.48

## Data Availability

The data will be made publicly available on NCBI GEO upon publication.

## Supplementary Materials

The following are available online at https://www.mdpi.com/2075-4426/11/3/175/s1, Figure S1: Change in FEV_1_ percent predicted in CAMP and SARP, Table S1: Genes in CAMP differentially expressed in corticosteroid poor responders compared to corticosteroid good responders, Table S2: Genes in SARP differentially expressed in corticosteroid poor responders compared to corticosteroid good responders, Table S3: Connectivity Map compounds with negative connectivity scores less than −50 in CAMP, Table S4: Connectivity Map compounds with negative connectivity scores less than −50 in SARP.

## Appendix A

SARP III RNA Sequencing: cDNA library was prepared with the Illumina TruSeq Stranded Total RNA (San Diego, CA, USA), quantified using qPCR and the PicoGreen dsDNA Assay Kit (Thermo Fisher Scientific, Waltham, MA, USA), and assessed for size and concentration using the 2100 Bioanalyzer System and 2200 TapeStation (Agilent, Santa Clara, CA, USA). Sequencing was performed on the Illumina HiSeq 2500 System with an average depth of 20 million reads. STAR was used to align the raw reads in sample FASTQ files to reference human genome version GRCh38 using default parameters after adapter sequence trimming by Skewer [56,57]. Raw counts for each mapped gene transcript were generated using the Bioconductor package Rsubread [58]. The counts were normalized using the Bioconductor package DESeq2 and log2-transformed [28]. A total of 65,988 gene transcripts in 38 samples (19 subjects) were generated. Gene transcripts with zero expression in all subjects were removed; 26,422 gene transcripts across both timepoints were retained. The data will be made publicly available on NCBI GEO upon publication.
