# Supplementary material for: Drug Repurposing to Treat Glucocorticoid Resistance in Asthma
Source: J Pers Med. 2021 Mar 3;11(3):175. doi: 10.3390/jpm11030175 (PMC7999884; doi:10.3390/jpm11030175)
Supplement: Supplementary file 1 [file jpm-11-00175-s001.pdf]

Journal of Personalized Medicine

Supplementary Materials

Drug repurposing to treat glucocorticoid resistance in asthma

Alberta L. Wang MD, MS, Ronald Panganiban PhD, Weiliang Qiu PhD, Alvin T. Kho PhD, Geoffrey Chupp MD, Deborah A. Meyers PhD, Eugene R. Bleecker MD, Scott T. Weiss MD, MS, Quan Lu PhD, Kelan G. Tantisira MD, MPH

Figure S1. Change in FEV_1 percent predicted in CAMP and SARP

a) Change in FEV_1 % predicted in CAMP from enrollment to month two on inhaled budesonide. Subjects in the first tertile were poor responders and in the third tertile were good responders. Median change in FEV_1 % predicted in the lowest tertile was -2.7 (± 6.7), middle tertile was 4.9 (± 3.3), and highest tertile was 15.8 (± 16.7) ($P < 0.001$).

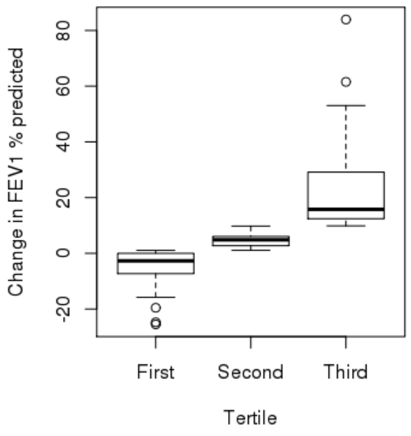

b) Change in FEV_1 % predicted in SARP from visit 2 prior to administration of intramuscular triamcinolone to visit 3 eighteen days post-corticosteroid administration. Median change in FEV_1 % predicted of -4.0 (± 5.6) in poor responders and 2.6 (± 3.9) in good responders ($P < 0.001$).

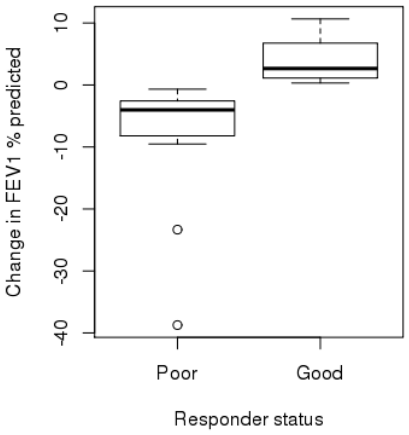

Table S1. Genes in CAMP differentially expressed in corticosteroid poor responders compared to corticosteroid good responders

Entrez gene ID	HGNC gene symbol	Chromosome band	T-statistic	P-value
23090	*ZNF423*	16q12.1	-4.18	6.17E-05
3763	*KCNJ6*	21q22.13	-4.16	6.81E-05
168002	*DACT2*	6q27	-3.86	1.99E-04
8704	*B4GALT2*	1p34.1	-3.76	2.91E-04
84570	*COL25A1*	4q25	-3.71	3.42E-04
51166	*AADAT*	4q33	3.66	4.12E-04
6785	*ELOVL4*	6q14.1	-3.63	4.57E-04
402317	*OR2A42*	7q35	-3.62	4.59E-04
9796	*PHYHIP*	8p21.3	3.57	5.50E-04
10107	*TRIM10*	6p22.1	-3.50	6.98E-04
54581	*SCAND2P*	15q25.2	-3.47	7.71E-04
200312	*RNF215*	22q12.2	3.47	7.74E-04
148170	*CDC42EP5*	19q13.42	-3.41	9.53E-04
92747	*BPIFB1*	20q11.21	-3.36	1.09E-03
153579	*BTNL9*	5q35.3	3.36	1.10E-03
172	*AFG3L1P*	16q24.3	3.33	1.23E-03
337963	*KRTAP23-1*	21q22.11	-3.33	1.23E-03
399879	NA	NA	-3.32	1.28E-03
391114	*OR6K3*	1q23.1	-3.31	1.31E-03
389337	*ARHGEF37*	5q32	3.27	1.46E-03
85317	*BAGE4*	21p11.1	-3.25	1.58E-03
9611	*NCOR1*	17p12-p11.2	-3.23	1.66E-03
84166	*NLRC5*	16q13	3.18	1.96E-03
284129	*SLC26A11*	17q25.3	3.18	1.99E-03
6728	*SRP19*	5q22.2	-3.17	2.04E-03
6121	*RPE65*	1p31.3	-3.16	2.07E-03
10347	*ABCA7*	19p13.3	3.16	2.10E-03
25907	*TMEM158*	3p21.31	-3.14	2.24E-03
178	*AGL*	1p21.2	-3.13	2.29E-03
10123	*ARL4C*	2q37.1	3.13	2.30E-03
341276	*OR10A2*	11p15.4	-3.12	2.34E-03
84645	*C22orf23*	22q13.1	-3.10	2.51E-03
5599	*MAPK8*	10q11.22	3.10	2.55E-03
8601	*RGS20*	8q11.23	3.08	2.67E-03
57795	*BRINP2*	1q25.2	-3.07	2.72E-03
149469	NA	NA	-3.06	2.86E-03
27239	*GPR162*	12p13.31	-3.05	2.92E-03
28981	*IFT81*	12q24.11	-3.05	2.95E-03

554226	*ANKRD30BL*	2q21.2	3.05	2.96E-03
504188	NA	NA	-3.05	2.96E-03
117144	*CATSPER1*	11q13.1	-3.04	3.00E-03
899	*CCNF*	16p13.3	-3.04	3.04E-03
5333	*PLCD1*	3p22.2	3.03	3.08E-03
56034	*PDGFC*	4q32.1	3.03	3.16E-03
6442	*SGCA*	17q21.33	-3.02	3.25E-03
10520	*ZNF211*	19q13.43	3.02	3.26E-03
56888	*KCMF1*	2p11.2	-3.00	3.39E-03
6772	*STAT1*	2q32.2	-2.98	3.58E-03
54819	*ZCCHC10*	5q31.1	-2.97	3.69E-03
30812	*SOX8*	16p13.3	-2.97	3.71E-03
11162	*NUDT6*	4q28.1	-2.96	3.82E-03
8793	*TNFRSF10D*	8p21.3	2.96	3.84E-03
256933	*NPB*	17q25.3	2.96	3.84E-03
9424	*KCNK6*	19q13.2	2.96	3.88E-03
9128	*PRPF4*	9q32	-2.95	3.99E-03
2115	*ETV1*	7p21.2	2.94	4.09E-03
51700	*CYB5R2*	11p15.4	-2.93	4.25E-03
9628	*RGS6*	14q24.2	-2.92	4.34E-03
84929	*FIBCD1*	9q34.12	2.91	4.50E-03
51816	*ADA2*	22q11.1	2.91	4.50E-03
160518	*DENND5B*	12p11.21	-2.91	4.50E-03
252995	*FNDC5*	1p35.1	-2.90	4.54E-03
4361	*MRE11*	11q21	2.89	4.71E-03
2230	*FDX1*	11q22.3	-2.88	4.80E-03
283685	*GOLGA6L2*	15q11.2	-2.88	4.81E-03
55867	*SLC22A11*	11q13.1	2.87	5.08E-03
337976	*KRTAP20-2*	21q22.11	2.86	5.12E-03
166336	*PRICKLE2*	3p14.1	2.86	5.14E-03
9609	*RAB36*	22q11.23	2.86	5.17E-03
23414	*ZFPM2*	8q23.1	2.86	5.20E-03
387758	*FIBIN*	11p14.2	2.85	5.26E-03
4916	*NTRK3*	15q25.3	2.84	5.41E-03
79642	*ARSJ*	4q26	-2.84	5.41E-03
7399	*USH2A*	1q41	2.84	5.50E-03
84171	*LOXL4*	10q24.2	2.83	5.57E-03
127254	*ERICH3*	1p31.1	-2.83	5.57E-03
2054	*STX2*	12q24.33	2.83	5.58E-03
80279	*CDK5RAP3*	17q21.32	2.82	5.80E-03
4192	*MDK*	11p11.2	2.82	5.86E-03
8706	*B3GALNT1*	3q26.1	2.80	6.16E-03

147409	*DSG4*	18q12.1	2.80	6.18E-03
255352	NA	NA	2.80	6.19E-03
3305	*HSPA1L*	6p21.33	-2.79	6.26E-03
90187	*EMILIN3*	20q12	2.79	6.37E-03
8484	*GALR3*	22q13.1	-2.78	6.43E-03
81833	*SPACA1*	6q15	2.78	6.56E-03
56606	*SLC2A9*	4p16.1	2.77	6.67E-03
9689	*BZW1*	2q33.1	-2.77	6.77E-03
441639	*OR9K2*	12q13.2	-2.77	6.78E-03
66037	*BOLL*	2q33.1	2.76	6.79E-03
221718	*LINC00518*	6p24.3	-2.76	6.95E-03
8218	*CLTCL1*	22q11.21	-2.74	7.24E-03
1761	*DMRT1*	9p24.3	2.74	7.25E-03
400110	*ANKRD20A19P*	13q12.12	-2.74	7.32E-03
55281	*TMEM140*	7q33	-2.73	7.40E-03
25948	*KBTBD2*	7p14.3	2.73	7.51E-03
54567	*DLL4*	15q15.1	2.73	7.51E-03
4009	*LMX1A*	1q23.3	2.72	7.63E-03
339175	*METTL2A*	17q23.2	-2.72	7.66E-03
2917	*GRM7*	3p26.1	-2.72	7.76E-03
400823	*FAM177B*	1q41	-2.72	7.80E-03
27237	*ARHGEF16*	1p36.32	2.71	7.87E-03
26952	*SMR3A*	4q13.3	-2.71	7.89E-03
8503	*PIK3R3*	1p34.1	2.71	8.01E-03
3855	*KRT7*	12q13.13	2.70	8.19E-03
84106	*PRAM1*	19p13.2	-2.70	8.20E-03
10413	*YAP1*	11q22.1	-2.70	8.26E-03
54887	*UHRF1BP1*	6p21.31	2.69	8.33E-03
440590	*ZYG11A*	1p32.3	2.69	8.34E-03
127064	*OR2T12*	1q44	-2.69	8.43E-03
554251	*FBXO48*	2p13.3	2.69	8.45E-03
27129	*HSPB7*	1p36.13	-2.69	8.48E-03
283358	*B4GALNT3*	12p13.33	2.69	8.48E-03
8785	*MATN4*	20q13.12	-2.68	8.60E-03
84251	*SGIP1*	1p31.3	2.68	8.60E-03
84220	*RGPD5*	2q13	-2.68	8.62E-03
284296	NA	NA	2.68	8.65E-03
53335	*BCL11A*	2p16.1	-2.68	8.67E-03
3886	*KRT35*	17q21.2	-2.68	8.71E-03
337880	*KRTAP11-1*	21q22.11	-2.68	8.73E-03
917	*CD3G*	11q23.3	-2.67	8.89E-03
947	*CD34*	1q32.2	-2.67	8.91E-03

3671	*ISLR*	15q24.1	-2.66	9.05E-03
163259	*DENND2C*	1p13.2	2.66	9.10E-03
149233	*IL23R*	1p31.3	2.66	9.13E-03
22981	*NINL*	20p11.21	-2.66	9.23E-03
4620	*MYH2*	17p13.1	2.65	9.40E-03
10575	*CCT4*	2p15	-2.64	9.56E-03
6183	*MRPS12*	19q13.2	-2.64	9.57E-03
196527	*ANO6*	12q12	-2.64	9.63E-03
388646	*GBP7*	1p22.2	2.63	9.91E-03
777	*CACNA1E*	1q25.3	2.63	9.94E-03
4197	NA	NA	2.63	9.94E-03
5803	*PTPRZ1*	7q31.32	2.63	9.97E-03

Table S2. Genes in SARP differentially expressed in corticosteroid poor responders compared to corticosteroid good responders

Ensembl stable ID	Entrez gene ID	HGNC gene symbol	Chromosome band	T-statistic	P-value
ENSG00000188242	170622	*COMMD6*	13q22.2	-4.75	2.19E-04
ENSG00000224936	100302691	*LINC00184*	1q42.3	-4.68	2.52E-04
ENSG00000172425	221421	*RSPH9*	6p21.1	-4.67	2.59E-04
ENSG00000196465	90576	*ZNF799*	19p13.2	-4.65	2.70E-04
ENSG00000260053	352963	*HLA-P*	6p22.1	-4.61	2.92E-04
ENSG00000162910	574407	*OBSCN-AS1*	1q42.13	-4.36	4.87E-04
ENSG00000158560	5207	*PFKFB1*	Xp11.21	-4.32	5.31E-04
ENSG00000213971	---	NA	NA	-4.24	6.25E-04
ENSG00000272888	---	NA	NA	4.21	6.65E-04
ENSG00000236035	100507114	*LINC00445*	13q13.3	-4.17	7.30E-04
ENSG00000239128	---	NA	NA	4.16	7.36E-04
ENSG00000248126	---	NA	NA	-4.15	7.52E-04
ENSG00000174951	170506	*DHX36*	3q25.2	-4.09	8.65E-04
ENSG00000213046	163486	*DENND1B*	1q31.3	4.07	8.88E-04
ENSG00000262516	---	NA	NA	-4.07	8.89E-04
ENSG00000163202	4184	*SMCP*	1q21.3	-4.02	9.90E-04
ENSG00000231169	---	NA	NA	-3.99	1.07E-03
ENSG00000273192	246785	*OLA1P1*	22q13.2	-3.97	1.11E-03
ENSG00000261519	284215	*DLGAP1-AS5*	18p11.31	-3.94	1.17E-03
ENSG00000167618	284339	*TMEM145*	19q13.2	-3.91	1.24E-03
ENSG00000187952	441194	*PMS2CL*	7p22.1	3.88	1.33E-03
ENSG00000131686	261734	*NPHP4*	1p36.31	-3.83	1.49E-03
ENSG00000152082	112714	*TUBA3E*	2q21.1	-3.79	1.60E-03
ENSG00000167311	10449	*ACAA2*	18q21.1	3.79	1.60E-03
ENSG00000088320	22974	*TPX2*	20q11.21	3.75	1.75E-03
ENSG00000271525	---	NA	NA	3.75	1.75E-03
ENSG00000207926	407028	*MIR302A*	4q25	-3.73	1.82E-03
ENSG00000105205	79935	*CCNP*	19q13.2	3.72	1.88E-03
ENSG00000212158	---	NA	NA	-3.70	1.96E-03
ENSG00000169918	2990	*GUSB*	7q11.21	3.70	1.97E-03
ENSG00000259208	---	NA	NA	-3.69	2.00E-03
ENSG00000073536	6389	*SDHA*	5p15.33	-3.66	2.12E-03
ENSG00000237211	---	NA	NA	3.64	2.22E-03
ENSG00000120279	80231	*TASL*	Xp21.2	3.62	2.29E-03
ENSG00000262096	---	NA	NA	-3.60	2.40E-03
ENSG00000164077	4486	*MST1R*	3p21.31	-3.60	2.42E-03
ENSG00000257838	---	NA	NA	-3.59	2.45E-03
ENSG00000140465	170691	*ADAMTS17*	15q26.3	-3.54	2.72E-03

ENSG00000183454	---	NA	NA	-3.51	2.89E-03
ENSG00000198838	11179	*ZNF277*	7q31.1	-3.51	2.89E-03
ENSG00000166741	116285	*ACSM1*	16p12.3	-3.49	3.04E-03
ENSG00000257246	---	NA	NA	-3.48	3.12E-03
ENSG00000250045	---	NA	NA	-3.47	3.17E-03
ENSG00000139610	6601	*SMARCC2*	12q13.2	-3.44	3.35E-03
ENSG00000164051	84126	*ATRIP*	3p21.31	-3.43	3.44E-03
ENSG00000229020	---	NA	NA	-3.43	3.44E-03
ENSG00000175782	145270	*PRIMA1*	14q32.12	-3.43	3.48E-03
ENSG00000230956	646366	*MAS1LP1*	6p22.1	3.42	3.51E-03
ENSG00000253146	---	NA	NA	-3.42	3.51E-03
ENSG00000250425	100130017	*FTLP10*	4q13.2	-3.42	3.53E-03
ENSG00000258452	79334	*OR11H2*	14q11.2	-3.41	3.61E-03
ENSG00000158220	55179	*FAIM*	3q22.3	-3.40	3.70E-03
ENSG00000161243	93099	*DMKN*	19q13.12	-3.39	3.73E-03
ENSG00000207650	407020	*MIR28*	3q28	3.35	4.10E-03
ENSG00000165192	57526	*PCDH19*	Xq22.1	-3.34	4.15E-03
ENSG00000136444	4836	*NMT1*	17q21.31	-3.34	4.16E-03
ENSG00000242683	3108	*HLA-DMA*	6p21.32	3.34	4.17E-03
ENSG00000218748	---	NA	NA	-3.33	4.24E-03
ENSG00000229754	---	NA	NA	3.32	4.36E-03
ENSG00000267317	---	NA	NA	-3.31	4.41E-03
ENSG00000160224	755	*CFAP410*	21q22.3	3.31	4.42E-03
ENSG00000158477	9825	*SPATA2*	20q13.13	3.31	4.42E-03
ENSG00000123989	23549	*DNPEP*	2q35	3.31	4.45E-03
ENSG00000249489	---	NA	NA	3.31	4.47E-03
ENSG00000276103	---	NA	NA	-3.31	4.47E-03
ENSG00000235817	441931	*VN1R17P*	1q44	-3.31	4.48E-03
ENSG00000204529	5460	*POU5F1*	6p21.33	-3.31	4.49E-03
ENSG00000235437	144832	*ESRRAP2*	13q12.11	3.30	4.51E-03
ENSG00000203782	574414	*PRR9*	1q21.3	3.30	4.55E-03
ENSG00000183248	4764	*NF1P3*	21q11.2	3.30	4.57E-03
ENSG00000087494	116154	*PHACTR3*	20q13.32-q13.33	3.29	4.59E-03
ENSG00000242282	100133100	*RPL6P8*	3q25.33	-3.29	4.61E-03
ENSG00000215199	389207	*GRXCR1*	4p13	3.29	4.66E-03
ENSG00000258212	---	NA	NA	-3.29	4.67E-03
ENSG00000242314	106479473	*RN7SL685P*	8q22.3	-3.28	4.77E-03
ENSG00000225828	2074	*ERCC6*	10q11.23	-3.27	4.80E-03
ENSG00000141753	60681	*FKBP10*	17q21.2	3.27	4.81E-03
ENSG00000225338	---	NA	NA	-3.27	4.81E-03
ENSG00000188747	440452	*TBC1D3P2*	17q23.2	-3.27	4.84E-03
ENSG00000272501	---	NA	NA	-3.25	5.01E-03

ENSG00000249852	222537	*HS3ST5*	6q21-q22.1	-3.25	5.08E-03
ENSG00000187024	337978	*KRTAP21-2*	21q22.11	3.24	5.12E-03
ENSG00000164400	23176	*SEPTIN8*	5q31.1	3.23	5.25E-03
ENSG00000243970	391559	*VTI1BP1*	3q12.1	-3.23	5.28E-03
ENSG00000211666	28802	*IGLV3-12*	22q11.22	3.22	5.34E-03
ENSG00000248578	---	NA	NA	-3.22	5.37E-03
ENSG00000244094	100128140	*RPS4XP17*	17p13.3	-3.21	5.44E-03
ENSG00000264017	---	NA	NA	-3.21	5.45E-03
ENSG00000218416	---	NA	NA	3.21	5.48E-03
ENSG00000228347	106479047	*EFCAB14P1*	1p34.3	-3.20	5.60E-03
ENSG00000271100	---	NA	NA	3.20	5.61E-03
ENSG00000117281	1889	*ECE1*	1p36.12	-3.20	5.63E-03
ENSG00000211592	28946	*IGKJ5*	2p11.2	3.19	5.67E-03
ENSG00000134443	57614	*RELCH*	18q21.33	3.19	5.72E-03
ENSG00000188825	84464	*SLX4*	16p13.3	3.18	5.80E-03
ENSG00000197121	6714	*SRC*	20q11.23	3.18	5.86E-03
ENSG00000145794	171019	*ADAMTS19*	5q23.3	3.17	5.90E-03
ENSG00000243466	27160	*INGX*	Xq13.1	3.16	6.04E-03
ENSG00000225470	---	NA	NA	3.16	6.11E-03
ENSG00000231378	100132467	*RNF2P1*	9p24.1	3.15	6.21E-03
ENSG00000212579	654321	*SNORA75*	2q37.1	-3.15	6.24E-03
ENSG00000265790	---	NA	NA	3.14	6.34E-03
ENSG00000278829	---	NA	NA	-3.14	6.37E-03
ENSG00000186453	400830	*DEFB132*	20p13	3.13	6.45E-03
ENSG00000233028	---	NA	NA	-3.13	6.45E-03
ENSG00000230841	---	NA	NA	-3.13	6.47E-03
ENSG00000211694	6983	*TRGV9*	7p14.1	3.13	6.48E-03
ENSG00000220161	---	NA	NA	-3.13	6.53E-03
ENSG00000225264	100506161	*TAF1A-AS1*	1q41	-3.13	6.53E-03
ENSG00000236723	---	NA	NA	-3.12	6.59E-03
ENSG00000228775	---	NA	NA	-3.12	6.63E-03
ENSG00000207975	693192	*MIR607*	10q24.1	-3.12	6.66E-03
ENSG00000230993	100462812	*FGFR3P1*	6p21.33	3.10	6.86E-03
ENSG00000224186	---	NA	NA	-3.10	6.88E-03
ENSG00000230280	101929589	*RPL7P61*	2q24.3	-3.10	6.93E-03
ENSG00000255107	---	NA	NA	-3.09	7.02E-03
ENSG00000197635	5275	*SERPINB13*	18q21.33	3.09	7.09E-03
ENSG00000149927	8479	*HIRIP3*	16p11.2	-3.09	7.10E-03
ENSG00000273142	---	NA	NA	3.08	7.18E-03
ENSG00000187860	164118	*TTC24*	1q22	-3.08	7.21E-03
ENSG00000275793	503841	*DEFB106B*	8p23.1	3.07	7.28E-03
ENSG00000172399	171024	*SYNPO2*	4q26	-3.07	7.31E-03

ENSG00000238460	100147762	*RNU7-14P*	20q13.2	-3.07	7.31E-03
ENSG00000144648	729085	*GASK1A*	3p22.1	3.07	7.40E-03
ENSG00000069018	375449	*MAST4*	5q12.3	3.07	7.43E-03
ENSG00000161682	80174	*DBF4B*	17q21.31	-3.06	7.48E-03
ENSG00000134339	63982	*ANO3*	11p14.3-p14.2	3.06	7.48E-03
ENSG00000089847	55760	*DHX32*	10q26.2	-3.06	7.50E-03
ENSG00000260442	---	NA	NA	3.06	7.52E-03
ENSG00000236269	10211	*FLOT1*	6p21.33	-3.05	7.59E-03
ENSG00000138135	10660	*LBX1*	10q24.32	-3.05	7.71E-03
ENSG00000101188	55257	*MRGBP*	20q13.33	-3.04	7.75E-03
ENSG00000239570	28881	*IGKV2D-30*	2p11.2	3.04	7.82E-03
ENSG00000105963	22797	*TFEC*	7q31.2	-3.04	7.82E-03
ENSG00000117481	50999	*TMED5*	1p22.1	-3.04	7.87E-03
ENSG00000086717	7462	*LAT2*	7q11.23	-3.04	7.88E-03
ENSG00000205238	26637	*OR7E36P*	13q14.11	-3.03	7.93E-03
ENSG00000184774	644538	*SMIM10*	Xq26.3	-3.03	8.04E-03
ENSG00000251136	646641	*RPL7L1P7*	3q23	-3.02	8.15E-03
ENSG00000145194	90113	*VWA5B2*	3q27.1	-3.02	8.15E-03
ENSG00000205037	93035	*PKHD1L1*	8q23.1-q23.2	-3.02	8.21E-03
ENSG00000030304	22807	*IKZF2*	2q34	-3.01	8.38E-03
ENSG00000254701	---	NA	NA	3.01	8.40E-03
ENSG00000156463	392255	*GDF6*	8q22.1	3.01	8.40E-03
ENSG00000114023	4968	*OGG1*	3p25.3	-3.01	8.41E-03
ENSG00000181513	---	NA	NA	3.00	8.42E-03
ENSG00000183549	399814	*C10orf120*	10q26.13	-3.00	8.49E-03
ENSG00000239983	106479377	*RN7SL420P*	1p13.1	-3.00	8.50E-03
ENSG00000136463	55852	*TEX2*	17q23.3	-3.00	8.53E-03
ENSG00000226491	51596	*CUTA*	6p21.32	-3.00	8.58E-03
ENSG00000228036	---	NA	NA	2.99	8.72E-03
ENSG00000188167	199777	*ZNF626*	19p12	-2.98	8.85E-03
ENSG00000114698	51409	*HEMK1*	3p21.31	2.98	8.88E-03
ENSG00000187800	65243	*ZFP69B*	1p34.2	2.98	8.89E-03
ENSG00000207627	723779	*MIR651*	Xp22.31	-2.98	8.95E-03
ENSG00000275882	---	NA	NA	-2.97	8.98E-03
ENSG00000217557	22815	*TDGF1P4*	6p25.2	2.96	9.30E-03
ENSG00000121057	8161	*COIL*	17q22	-2.96	9.31E-03
ENSG00000228192	100462876	*ELOCP14*	Yq11.222	-2.95	9.35E-03
ENSG00000226800	100874388	*OSTCP8*	9p13.3	-2.95	9.38E-03
ENSG00000090776	55066	*PDPR*	16q22.1	-2.95	9.38E-03
ENSG00000267691	163088	*TAF9P3*	19q12	-2.95	9.47E-03
ENSG00000229608	106146146	*LINC01079*	13q12.2	-2.94	9.55E-03
ENSG00000137040	64425	*POLR1E*	9p13.2	2.94	9.56E-03

ENSG00000113966	27031	*NPHP3*	3q22.1	2.94	9.57E-03
ENSG00000216802	---	NA	NA	-2.94	9.59E-03
ENSG00000235514	106480731	*C6orf47-AS1*	6p21.33	-2.94	9.60E-03
ENSG00000168061	116071	*BATF2*	11q13.1	-2.94	9.63E-03
ENSG00000264994	---	NA	NA	-2.94	9.63E-03
ENSG00000163519	2199	*FBLN2*	3p25.1	-2.94	9.73E-03
ENSG00000227972	728758	*PIN4P1*	15q15.3	2.93	9.75E-03
ENSG00000155090	5799	*PTPRN2*	7q36.3	2.93	9.77E-03
ENSG00000196860	285190	*RGPD4*	2q12.3	2.93	9.79E-03
ENSG00000165868	414241	*SHLD2P1*	10q11.22	2.93	9.87E-03
ENSG00000166246	79827	*CLMP*	11q24.1	2.93	9.88E-03
ENSG00000264311	106481853	*RN7SL644P*	10q23.33	2.93	9.89E-03
ENSG00000188428	---	NA	NA	2.92	9.96E-03
ENSG00000026652	57823	*SLAMF7*	1q23.3	-2.92	9.96E-03

Table S3. Connectivity Map compounds with negative connectivity scores less than -50 in CAMP

Score	Broad ID	Name	Description
-50.25	BRD-A81233518	glycopyrrolate	Acetylcholine receptor antagonist
-50.42	BRD-A38749782	fludrocortisone	Glucocorticoid receptor agonist
-50.53	BRD-K37206356	rhamnetin	HDAC inhibitor
-50.67	BRD-A65767837	hydrocortisone	Glucocorticoid receptor agonist
-50.69	BRD-K45401373	betulinic-acid	Apoptosis stimulant
-51.30	BRD-K92778217	mefenamic-acid	Cyclooxygenase inhibitor
-51.38	BRD-A41722204	sulmazole	Adenosine receptor antagonist
-51.72	BRD-K46384212	o-3M3FBS	phospholipase activator
-51.73	BRD-M45964048	verteporfin	Photosensitizing agent
-51.73	BRD-A01346607	flumetasone	Glucocorticoid receptor agonist
-51.98	BRD-K87919739	tyrphostin-AG-825	Receptor tyrosine protein kinase inhibitor
-52.13	BRD-K97810537	beclometasone	Glucocorticoid receptor agonist
-52.16	BRD-K86887724	dofetilide	Potassium channel blocker
-52.31	BRD-K59469039	AG-879	Angiogenesis inhibitor
-52.33	BRD-K86958018	olvanil	TRPV agonist
-52.50	BRD-A96255180	ribavirin	Antiviral
-52.83	BRD-K05464208	JX-401	p38 MAPK inhibitor
-52.97	BRD-A04756508	norgestimate	Progesterone receptor agonist
-53.06	BRD-K92301463	16,16-dimethylprostaglandin-e2	Prostanoid receptor agonist
-53.25	BRD-A03623303	metoprolol	Adrenergic receptor antagonist
-53.43	BRD-A51182606	chloramphenicol	Protein synthesis inhibitor
-53.57	BRD-A04352665	maraviroc	CC chemokine receptor antagonist
-53.63	BRD-K70557564	zosuquidar	P-glycoprotein inhibitor
-53.74	BRD-A72988804	tiaprofenic-acid	Cyclooxygenase inhibitor
-53.84	BRD-A68723818	brompheniramine	Histamine receptor antagonist
-54.16	BRD-K69688083	mestinon	Cholinesterase inhibitor

-54.44	BRD-K17674993	diflorasone	Corticosteroid agonist
-54.46	BRD-A65597028	RX-821002	Adrenergic receptor antagonist
-54.53	BRD-A65550283	ginsenoside	Steroid hormone receptor agonist
-54.68	BRD-K74430258	1,2-dichlorobenzene	Hepatotoxicant
-55.04	BRD-K71926323	marbofloxacin	Bacterial DNA gyrase inhibitor
-55.18	BRD-K68873215	phosphodiesterase-V-inhibitor-II	Phosphodiesterase inhibitor
-55.23	BRD-K37792168	denbufylline	Phosphodiesterase inhibitor
-55.40	BRD-A55594068	vinblastine	Microtubule inhibitor
-55.81	BRD-K53318339	vinpocetine	Phosphodiesterase inhibitor
-55.87	BRD-K87158025	benzamil	Sodium channel blocker
-55.96	BRD-K97056771	HY-11007	BCR-ABL kinase inhibitor
-55.99	BRD-A25067867	benzatropine	Acetylcholine receptor antagonist
-56.03	BRD-K77998258	ganglioside	SRC activator
-56.28	BRD-K23583188	lavendustin-a	EGFR inhibitor
-56.48	BRD-A17065207	brefeldin-a	Protein synthesis inhibitor
-56.49	BRD-K21565985	xylazine	Adrenergic receptor agonist
-56.50	BRD-A75935363	atracurium	Acetylcholine receptor antagonist
-57.04	BRD-A39522003	OMDM-2	FAAH inhibitor
-57.17	BRD-K70241288	L-692585	Growth hormone releasing peptide ligand agonist
-57.19	BRD-A69960130	bromocriptine	Dopamine receptor agonist
-57.34	BRD-K11634954	GBR-13069	Dopamine uptake inhibitor
-57.39	BRD-A15034104	bergenin	Interleukin inhibitor
-57.45	BRD-K41868777	W-5	Calmodulin antagonist
-57.64	BRD-A43974499	reboxetine	Adrenergic receptor antagonist
-57.66	BRD-K95885906	quercetagetin	PIM inhibitor
-57.70	BRD-A16332958	modafinil	Adrenergic receptor agonist
-57.95	BRD-K63874012	thioperamide	Histamine receptor antagonist
-58.13	BRD-K75532464	FTI-276	Farnesyltransferase inhibitor

-58.36	BRD-K37848908	ceforanide	Penicillin binding protein inhibitor
-58.41	BRD-K18787491	U-0126	MEK inhibitor
-58.66	BRD-K62056274	quipazine	Serotonin receptor agonist
-58.77	BRD-K67445247	flurofamide	Urease inhibitor
-58.89	BRD-K82561139	ricinine	Casein kinase inhibitor
-58.98	BRD-A13946108	sulindac	Cyclooxygenase inhibitor
-58.98	BRD-A92630576	trimebutine	Opioid receptor agonist
-58.99	BRD-A89175223	bisoprolol	Adrenergic receptor antagonist
-59.28	BRD-K49372556	mofezolac	Cyclooxygenase inhibitor
-59.30	BRD-K10176267	L-701252	Glutamate receptor antagonist
-59.37	BRD-K00675675	CL-82198	Metalloproteinase inhibitor
-59.41	BRD-K04111260	raclopride	Dopamine receptor antagonist
-59.58	BRD-K06208435	YS-035	Calcium channel blocker
-59.67	BRD-K43764301	dexketoprofen	Cyclooxygenase inhibitor
-59.72	BRD-K63533170	AM-630	Cannabinoid receptor antagonist
-59.94	BRD-K85383046	IAA-94	Chloride channel blocker
-59.95	BRD-A96897502	U-74389F	Lipid peroxidase inhibitor
-60.01	BRD-K13664374	dichloroacetic-acid	Pyruvate dehydrogenase kinase inhibitor
-60.05	BRD-K54708045	nTZDpa	PPAR receptor agonist
-60.18	BRD-K53857191	risperidone	Dopamine receptor antagonist
-60.28	BRD-K48869804	icilin	TRPV agonist
-60.56	BRD-K05181084	NGB-2904	Dopamine receptor antagonist
-60.67	BRD-K43860855	iobenguane	Antineoplastic
-60.76	BRD-K11129031	gemfibrozil	Lipoprotein lipase activator
-60.86	BRD-K49945136	GR-113808	Serotonin receptor antagonist
-60.92	BRD-K81729199	AQ-RA741	Acetylcholine receptor antagonist
-61.00	BRD-K40619305	larixinic-acid	Compound that interacts with metal centers
-61.37	BRD-K11801786	trimidox	Ribonucleotide reductase inhibitor

-61.37	BRD-A89337244	PD-102807	Acetylcholine receptor antagonist
-61.46	BRD-A29644307	nomifensine	Dopamine uptake inhibitor
-61.63	BRD-K03600606	catechin	Beta secretase inhibitor
-61.73	BRD-K36965586	m-chlorophenylbiguanide	Serotonin receptor agonist
-61.75	BRD-A67799922	phenoxybenzamine	Adrenergic receptor antagonist
-61.93	BRD-K60770992	pergolide	Dopamine receptor agonist
-62.03	BRD-A90799790	isradipine	Calcium channel blocker
-62.35	BRD-K07220430	cinnarizine	Calcium channel blocker
-62.36	BRD-K39733634	L-161982	Prostanoid receptor antagonist
-62.83	BRD-K27351809	nomegestrol	Progesterone receptor agonist
-62.97	BRD-K01095011	finasteride	5-alpha reductase inhibitor
-63.46	BRD-A41555725	chlortetracycline	Protein synthesis inhibitor
-63.63	BRD-A35108200	dexamethasone	Glucocorticoid receptor agonist
-63.65	BRD-K92731339	perindopril	ACE inhibitor
-63.78	BRD-K84266862	BRL-50481	Phosphodiesterase inhibitor
-63.85	BRD-A98299281	velnacrine	cholinesterase inhibitor
-63.90	BRD-A45140972	meclocycline	Bacterial 30S ribosomal subunit inhibitor
-64.10	BRD-U97083655	teicoplanin	Bacterial cell wall synthesis inhibitor
-64.13	BRD-K91696562	orantinib	FGFR inhibitor
-64.20	BRD-K62982419	cilomilast	Phosphodiesterase inhibitor
-64.30	BRD-K41170226	deoxycholic-acid	G protein-coupled receptor agonist
-64.59	BRD-K61401890	deguelin	NADH-ubiquinone oxidoreductase (Complex I) inhibitor
-64.63	BRD-K09859624	methantheline	Acetylcholine receptor antagonist
-64.90	BRD-K32318651	acyclovir	DNA polymerase inhibitor
-64.91	BRD-A15131297	benazepril	ACE inhibitor
-65.03	BRD-K43796186	benzyl-quinazolin-4-yl-amine	EGFR inhibitor
-65.27	BRD-K01253243	SB-590885	RAF inhibitor
-65.34	BRD-K62996583	lidoflazine	Calcium channel blocker

-65.63	BRD-A79314293	cephalosporanic-acid	Bacterial cell wall synthesis inhibitor
-65.92	BRD-K69328504	L-690488	Inositol monophosphatase inhibitor
-66.01	BRD-K56064827	EI-273	PKC inhibitor
-66.20	BRD-K02123250	JNJ-38877605	Tyrosine kinase inhibitor
-66.87	BRD-A07780951	orciprenaline	Adrenergic receptor agonist
-67.02	BRD-K76908866	CP-724714	EGFR inhibitor
-67.07	BRD-K81209159	herniarin	Acetylcholinesterase inhibitor
-67.08	BRD-K63151507	MNITMT	Lymphocyte inhibitor
-67.10	BRD-A32164164	methyllycaconitine	Acetylcholine receptor antagonist
-67.11	BRD-K28143534	cyproheptadine	Histamine receptor antagonist
-67.52	BRD-A31312900	montelukast	Leukotriene receptor antagonist
-67.75	BRD-A97104540	fenoterol	Adrenergic receptor agonist
-68.07	BRD-K93188295	ARC-239	Adrenergic receptor antagonist
-68.27	BRD-K46424862	hymecromone	Monoamine oxidase inhibitor
-68.44	BRD-K29359156	ebselen	H+/K+-ATPase inhibitor
-68.48	BRD-K26573499	DMAB-anabaseine	Adrenergic receptor agonist
-68.50	BRD-K89152108	liothyronine	Thyroid hormone stimulant
-68.73	BRD-A99411506	esculin	Antioxidant
-68.92	BRD-K88868628	iodoacetic-acid	Cysteine peptidase inhibitor
-68.97	BRD-K14681867	somatostatin	Somatostatin receptor agonist
-69.16	BRD-A12016240	LY-278584	Serotonin receptor antagonist
-69.53	BRD-K96319534	phentermine	Dopamine uptake inhibitor
-69.56	BRD-K00184207	GR-206	Aryl hydrocarbon receptor ligand
-69.60	BRD-A49765801	fludroxycortide	Glucocorticoid receptor agonist
-69.61	BRD-K94887716	TFMPP	Serotonin receptor agonist
-70.20	BRD-K81473089	tacrine	Acetylcholinesterase inhibitor
-70.41	BRD-A12560204	nitrendipine	Calcium channel blocker
-70.52	BRD-A85280935	quinpirole	Dopamine receptor agonist

-70.69	BRD-A27143604	DPN	Estrogen receptor agonist
-70.78	BRD-K02867583	minaprine	Serotonin reuptake inhibitor
-71.26	BRD-K66707493	lawsone	Coloring agent
-71.37	BRD-K32526544	DCEBIO	Potassium channel activator
-71.68	BRD-K67680372	CI-966	GAT inhibitor
-72.04	BRD-K07881437	danusertib	Aurora kinase inhibitor
-72.31	BRD-K09963420	saquinavir	HIV protease inhibitor
-72.33	BRD-K13927029	retinol	Retinoid receptor ligand
-72.42	BRD-K73109821	diazoxide	Potassium channel activator
-72.47	BRD-K34441861	moexipril	ACE inhibitor
-72.52	BRD-K16508793	diazepam	Benzodiazepine receptor agonist
-72.55	BRD-K42452249	EO-1428	p38 MAPK inhibitor
-72.61	BRD-K09635134	l-erythro-MAPP	negative control for D-erythro-MAPP
-72.67	BRD-K86727142	embelin	HCV inhibitor
-72.68	BRD-K01436366	XMD-1150	Leucine rich repeat kinase inhibitor
-72.9	BRD-K88568253	iproniazid	Monoamine oxidase inhibitor
-73.00	BRD-K40990712	hexamethyleneamiloride	Sodium/hydrogen antiport inhibitor
-73.00	BRD-K46766488	S-14506	Serotonin receptor agonist
-73.17	BRD-A25234499	aminoglutethimide	Glucocorticoid receptor antagonist
-73.74	BRD-A93255169	thalidomide	TNF production inhibitor
-74.22	BRD-K60762818	desipramine	Tricyclic antidepressant
-74.25	BRD-K14965640	ibuprofen	Cyclooxygenase inhibitor
-74.30	BRD-A31159102	fluoxetine	Selective serotonin reuptake inhibitor (SSRI)
-74.51	BRD-K25310650	ormetoprim	Bacterial antifolate
-74.67	BRD-A46179541	doxapram	Potassium channel blocker
-74.89	BRD-A00267231	hemado	Adenosine receptor agonist
-75.20	BRD-K26548821	quinpirole	Dopamine receptor agonist
-76.57	BRD-K52394958	GR-159897	Tachykinin antagonist

-76.74	BRD-A10903566	imiloxan	Adrenergic receptor antagonist
-76.77	BRD-K23204545	busulfan	DNA inhibitor
-77.09	BRD-A00993607	alprenolol	Adrenergic receptor antagonist
-77.11	BRD-K53561341	KIN001-220	Aurora kinase inhibitor
-77.32	BRD-K14643723	4-(2-Amino-ethyl)-benzenesulfonamide	carbonic anhydrase inhibitor
-77.35	BRD-K65814004	diphenyleneiodonium	Nitric oxide synthase inhibitor
-77.35	BRD-U94846492	quinine	Hemozoin biocrystallization inhibitor
-77.37	BRD-K66944906	fraxidin	Carbonic anhydrase inhibitor
-77.53	BRD-K38305202	domperidone	Dopamine receptor antagonist
-77.55	BRD-K39569857	avrainvillamide-analog-3	nucleophosmin inhibitor
-77.70	BRD-K39339537	epirizole	Cyclooxygenase inhibitor
-77.73	BRD-K58299615	RO-90-7501	Beta amyloid inhibitor
-78.56	BRD-K33882852	ZK-93423	Benzodiazepine receptor agonist
-78.72	BRD-K78485176	olmesartan	Angiotensin receptor antagonist
-78.82	BRD-K85133207	HDAC1-selective	HDAC inhibitor
-78.83	BRD-K02404261	caffeine	Adenosine receptor antagonist
-78.90	BRD-K43330982	JTE-013	Lysophospholipid receptor antagonist
-78.98	BRD-K26669427	WR-216174	PFMRK inhibitor
-79.02	BRD-A27489425	rolitetracycline	Bacterial 30S ribosomal subunit inhibitor
-79.32	BRD-K71035033	masitinib	KIT inhibitor
-79.35	BRD-K40227168	vinburnine	Adrenergic receptor antagonist
-79.38	BRD-K54210043	NS-1619	Calcium channel activator
-79.52	BRD-K99595596	salsolinol	Monoamine oxidase inhibitor
-79.60	BRD-A75455249	kavain	Calcium channel modulator
-79.72	BRD-A95696066	nisoxetine	Norepinephrine reuptake inhibitor
-79.74	BRD-K28761384	zuclopenthixol	Dopamine receptor antagonist
-79.74	BRD-K15791587	L-733060	Tachykinin antagonist
-79.93	BRD-A80638690	floxuridine	DNA synthesis inhibitor

-80.22	BRD-K67043667	altretamine	DNA synthesis inhibitor
-80.33	BRD-K08109516	L-701324	Glutamate receptor antagonist
-80.62	BRD-A15297126	fluocinonide	Glucocorticoid receptor agonist
-80.76	BRD-K21733600	rofecoxib	Cyclooxygenase inhibitor
-81.34	BRD-K53913732	SB-408124	Orexin receptor antagonist
-81.63	BRD-A33447119	oxfendazole	Anthelmintic
-81.67	BRD-A26711594	nicardipine	Calcium channel blocker
-81.93	BRD-K70327191	benzoxiquine	Anti-infective
-82.06	BRD-K29173907	isoflupredone	Glucocorticoid receptor agonist
-82.26	BRD-K90543092	levonorgestrel	Estrogen receptor agonist
-82.65	BRD-A34706053	CGP-12177	Adrenergic receptor agonist
-82.85	BRD-K28667793	pyrazinamide	Fatty acid synthase inhibitor
-83.10	BRD-A79237180	ascorbic-acid	Antioxidant
-83.72	BRD-K82983861	GW-0742	PPAR receptor agonist
-84.17	BRD-K30743633	TCPOBOP	CAR agonist
-84.20	BRD-K57631554	aminolevulinic-acid	Oxidizing agent
-84.35	BRD-K25224017	pirenperone	Serotonin receptor antagonist
-84.56	BRD-A16665823	butoconazole	Bacterial cell wall synthesis inhibitor
-84.81	BRD-K75641298	metoclopramide	Dopamine receptor antagonist
-85.00	BRD-A65013509	oxybutynin	Acetylcholine receptor antagonist
-86.00	BRD-A83326220	brazilin	Nitric oxide production inhibitor
-86.28	BRD-K70505054	ranitidine	Histamine receptor antagonist
-86.41	BRD-K71860425	CDK2-5-inhibitor	CDK inhibitor
-86.52	BRD-A87719232	naproxen	Cyclooxygenase inhibitor
-86.59	BRD-A73741725	exemestane	Aromatase inhibitor
-86.81	BRD-K74913225	brinzolamide	Carbonic anhydrase inhibitor
-87.45	BRD-K33308633	INCA-6	Calcineurin inhibitor
-87.89	BRD-K50388907	fenofibrate	PPAR receptor agonist

-87.89	BRD-K65331431	retinyl	vitamin analog
-88.17	BRD-K45435259	SCH-23390	Dopamine receptor antagonist
-88.38	BRD-K12994359	valdecoxib	Cyclooxygenase inhibitor
-88.58	BRD-K12260308	xanthoxyline	Antifungal
-88.76	BRD-A16478930	amcinonide	Glucocorticoid receptor agonist
-89.36	BRD-K63979671	etifenin	Compound used in hepatobiliary scans of the liver
-89.39	BRD-K02590140	O-2050	Cannabinoid receptor antagonist
-90.07	BRD-A55393291	testosterone	Androgen receptor agonist
-90.08	BRD-K32107296	temozolomide	DNA alkylating agent
-90.33	BRD-A94543220	bifonazole	Sterol demethylase inhibitor
-90.39	BRD-K19533706	tranilast	Angiogenesis inhibitor
-90.55	BRD-K76064317	tyrphostin-AG-1296	FLT3 inhibitor
-90.68	BRD-A62035778	scopolamine	Acetylcholine receptor antagonist
-90.70	BRD-K44899736	RO-16-6941	Monoamine oxidase inhibitor
-90.71	BRD-A91866971	SQ-29548	Thromboxane receptor antagonist
-90.92	BRD-K81521265	dicyclohexylurea	Epoxide hydolase inhibitor
-91.07	BRD-K37618799	MRS-1220	Adenosine receptor antagonist
-91.92	BRD-K39391626	ethylestrenol	Progesterone receptor agonist
-92.17	BRD-K78692225	leflunomide	Dihydroorotate dehydrogenase inhibitor
-92.65	BRD-K32645441	dipropyl-5ct	Serotonin receptor agonist
-92.76	BRD-A03216249	mepivacaine	Potassium channel blocker
-92.78	BRD-K91601245	mercaptopurine	Immunosuppressant
-93.01	BRD-K10177585	PSB-11	Adenosine receptor antagonist
-93.45	BRD-A07395371	esmolol	Adrenergic receptor antagonist
-93.52	BRD-K87991767	umbelliferone	Cyclooxygenase inhibitor
-93.70	BRD-K27141178	SB-203186	Serotonin receptor antagonist
-94.11	BRD-A35338386	NECA	Adenosine receptor agonist
-94.68	BRD-K51751936	alfadolone	GABA receptor agonist

-94.78	BRD-K18059238	gamma-linolenic-acid	Anti-inflammatory omega-6 fatty acid
-94.87	BRD-A64227845	SKF-77434	Dopamine receptor agonist
-95.00	BRD-K68756823	FR-180204	Selective ERK inhibitor
-95.07	BRD-K01555864	dibenzoylmethane	Antineoplastic
-95.36	BRD-K34098590	tienilic-acid	Sodium/potassium/chloride transporter inhibitor
-95.67	BRD-K64514229	toltrazuril	Antiprotozoal
-95.68	BRD-K67102207	phenylbutyrate	HDAC inhibitor
-95.68	BRD-K01648091	LE-300	Dopamine receptor antagonist
-96.08	BRD-K96037667	norethindrone	Progesterone receptor agonist
-96.19	BRD-A87606379	nadolol	Adrenergic receptor antagonist
-96.43	BRD-K59633790	VU-0420363-1	SARS coronavirus 3C-like protease inhibitor
-96.51	BRD-K76534306	enrofloxacin	Bacterial DNA gyrase inhibitor
-96.72	BRD-K49890030	gavestinel	Glutamate receptor antagonist
-96.81	BRD-K06712146	YM-90709	IL5 inhibitor
-97.07	BRD-A01826957	xanthinol	Vasodilator
-97.18	BRD-A41833852	naloxone	Opioid receptor antagonist
-97.50	BRD-K54529596	captopril	ACE inhibitor
-97.60	BRD-A16754160	ampicillin	Bacterial cell wall synthesis inhibitor
-97.79	BRD-K64310881	MW-STK33-3B	Potassium channel activator
-97.82	BRD-K97509413	coumestrol	Estrogen receptor agonist
-97.98	BRD-K90864987	cobalt(II)-chloride	HSP inducer
-98.32	BRD-K82941592	rosuvastatin	HMGCR inhibitor
-98.57	BRD-A02176148	tubaic-acid	Mitochondrial complex I inhibitor
-98.70	BRD-A04308630	genipin	Choleretic agent
-99.12	BRD-K79602928	metformin	Insulin sensitizer
-99.31	BRD-A71262238	nafadotride	Dopamine receptor antagonist
-99.61	BRD-K10466330	AVA	Nucleophosmin inhibitor

Table S4. Connectivity Map compounds with negative connectivity scores less than -50 in SARP

Score	Broad ID	Name	Description
-50.08	BRD-A26095496	clobetasol	Glucocorticoid receptor agonist
-50.46	BRD-K74236984	UNC-0321	Histone lysine methyltransferase inhibitor
-50.48	BRD-K17896185	FIT	Opioid receptor agonist
-50.57	BRD-K10670311	sulfasalazine	Antirheumatic
-50.65	BRD-K47278471	diphenhydramine	Histamine receptor antagonist
-50.65	BRD-M64432851	sunitinib	FLT3 inhibitor
-50.92	BRD-K43764301	dexketoprofen	Cyclooxygenase inhibitor
-50.97	BRD-A23637604	oxymetholone	Androgen receptor agonist
-51.10	BRD-K36740062	GSK-1070916	Aurora kinase inhibitor
-51.25	BRD-K68190965	GR-46611	Serotonin receptor agonist
-51.29	BRD-K63630713	etacrynic-acid	Sodium/potassium/chloride transporter inhibitor
-51.34	BRD-K26573499	DMAB-anabaseine	Adrenergic receptor agonist
-51.42	BRD-K88304388	dextrorphan	Glutamate receptor antagonist
-51.47	BRD-K26997899	SA-792574	Microtubule inhibitor
-51.70	BRD-K71926323	marbofloxacin	Bacterial DNA gyrase inhibitor
-51.96	BRD-K24201553	SB-269970	Serotonin receptor antagonist
-52.10	BRD-A12560204	nitrendipine	Calcium channel blocker
-52.28	BRD-K43978949	PIT	Purinergic receptor antagonist
-52.58	BRD-K71534238	GW-9508	Free fatty acid receptor agonist
-52.87	BRD-A36267905	buphenine	Adrenergic receptor agonist
-52.93	BRD-A62809825	thapsigargin	ATPase inhibitor
-53.08	BRD-K93480852	KN-93	Calcium-calmodulin dependent protein kinase inhibitor
-53.26	BRD-A38747044	KU-14R	Imidazoline receptor ligand
-53.29	BRD-K42142750	retrorsine	Antimitotic
-53.38	BRD-K86873305	piperacillin	Bacterial cell wall synthesis inhibitor
-53.39	BRD-A01295252	trans-7-hydroxy-pipat	Dopamine receptor ligand

-53.46	BRD-K30697463	desoximetasone	Glucocorticoid receptor agonist
-53.56	BRD-A04327189	synephrine	Adrenergic receptor agonist
-53.74	BRD-K12513978	fenbufen	Cyclooxygenase inhibitor
-53.74	BRD-K97764662	PD-173074	FGFR inhibitor
-54.17	BRD-K54987996	CAY-10578	Casein kinase inhibitor
-54.25	BRD-A41692738	TGX-221	PI3K inhibitor
-54.39	BRD-K88358234	xaliproden	Serotonin receptor agonist
-54.45	BRD-K09778810	FGIN-1-27	Inositol monophosphatase inhibitor
-54.50	BRD-K40578143	GR-79236	Adenosine receptor agonist
-54.69	BRD-K92138166	mammea-a	other antibiotic
-54.75	BRD-K37194137	III606050	Cytochrome P450 inhibitor
-54.91	BRD-K24526313	levcromakalim	Potassium channel activator
-54.92	BRD-A82238138	budesonide	Glucocorticoid receptor agonist
-54.97	BRD-A61470182	n-formylmethionylalanine	macrophage activator
-55.04	BRD-K45988865	tetramethylsilane	Internal standard for NMR spectroscopy
-55.04	BRD-K59637651	NSC-119889	Protein synthesis inhibitor
-55.26	BRD-A34751532	homosalate	HSP inducer
-55.62	BRD-A77050075	heraclenol	Vitamin K antagonist
-55.67	BRD-A73859745	glycodeoxycholic-acid	Apoptosis stimulant
-55.76	BRD-A13946108	sulindac	Cyclooxygenase inhibitor
-55.83	BRD-A62021152	WAY-161503	Serotonin receptor agonist
-55.85	BRD-U37049823	HG-6-64-01	RAF inhibitor
-56.02	BRD-A72441487	stiripentol	GABA uptake inhibitor
-56.12	BRD-K51816706	oxindole-I	VEGFR inhibitor
-56.12	BRD-U97083655	teicoplanin	Bacterial cell wall synthesis inhibitor
-56.22	BRD-K77008974	WYE-354	MTOR inhibitor
-56.43	BRD-K56403959	ZK-756326	CC chemokine receptor ligand
-56.45	BRD-A71203467	l-stepholidine	Dopamine receptor antagonist

-56.59	BRD-A16754160	ampicillin	Bacterial cell wall synthesis inhibitor
-56.65	BRD-K22662435	ganciclovir	DNA polymerase inhibitor
-56.83	BRD-K02130563	panobinostat	HDAC inhibitor
-56.89	BRD-K03642198	AY-9944	Hedgehog pathway modulator
-56.90	BRD-K18036262	L-168049	Glucagon receptor antagonist
-56.97	BRD-K05181463	L-741626	Dopamine receptor antagonist
-57.23	BRD-K57886322	fluocinonide	Glucocorticoid receptor agonist
-57.38	BRD-A62182663	YK-4279	Apoptosis stimulant
-57.45	BRD-A93255169	thalidomide	TNF production inhibitor
-58.07	BRD-A39522003	OMDM-2	FAAH inhibitor
-58.22	BRD-K35483542	alitretinoin	Retinoid receptor agonist
-58.49	BRD-K63195589	tipifarnib	Farnesyltransferase inhibitor
-58.59	BRD-K17075857	chloroxine	Opioid receptor antagonist
-58.68	BRD-A49765801	fludroxycortide	Glucocorticoid receptor agonist
-58.68	BRD-K95655893	MAZ-51	VEGFR inhibitor
-58.71	BRD-K91145395	prostratin	PKC activator
-59.04	BRD-K21450440	benzthiazide	Carbonic anhydrase inhibitor
-59.29	BRD-K36009368	NNC-63-0532	Opioid receptor agonist
-59.37	BRD-K26429091	J-104129	Acetylcholine receptor antagonist
-59.40	BRD-K74501079	azithromycin	Bacterial 50S ribosomal subunit inhibitor
-59.71	BRD-K01253243	SB-590885	RAF inhibitor
-59.99	BRD-K06159959	CCMQ	Inhibitor of the binding of homoquinolinic acid to non-NMDA sensitive sites
-60.05	BRD-K53959060	indirubin	CDK inhibitor
-60.31	BRD-K76723084	isotretinoin	Retinoid receptor agonist
-60.38	BRD-A31227688	kynuramine	Aryl hydrocarbon receptor activator
-60.82	BRD-K56957086	dacinostat	HDAC inhibitor
-61.00	BRD-A01593789	chlormadinone	5-alpha reductase inhibitor
-61.02	BRD-K01567962	pyrazolanthrone	JNK inhibitor

-61.10	BRD-K67578145	GDC-0879	RAF inhibitor
-61.12	BRD-A46179541	doxapram	Potassium channel blocker
-61.17	BRD-A89337244	PD-102807	Acetylcholine receptor antagonist
-61.48	BRD-K12502280	TG-101348	FLT3 inhibitor
-61.75	BRD-A28422058	L-689560	Glutamate receptor antagonist
-61.77	BRD-K08547377	irinotecan	Topoisomerase inhibitor
-61.79	BRD-A37837077	cyclazosin	Adrenergic receptor antagonist
-61.95	BRD-K75699339	rizatriptan	Serotonin receptor agonist
-62.01	BRD-A06390036	hydroquinidine	Antiarrhythmic
-62.17	BRD-A18917088	estradiol	Contraceptive agent
-62.21	BRD-K47869605	podophyllotoxin	Microtubule inhibitor
-62.39	BRD-K29173907	isoflupredone	Glucocorticoid receptor agonist
-62.55	BRD-K71799949	carbamazepine	Carboxamide antiepileptic
-62.75	BRD-K35458079	edaravone	Nootropic agent
-63.52	BRD-A25576662	streptozotocin	DNA alkylating agent
-63.89	BRD-K34330170	rotenonic-acid	Retinoid receptor antagonist
-64.22	BRD-K85925969	zalcitabine	Nucleoside reverse transcriptase inhibitor
-64.26	BRD-K19284129	salvinorin-a	Opioid receptor agonist
-64.27	BRD-K68202742	trichostatin-a	HDAC inhibitor
-64.58	BRD-A41995253	brucine	Glycine receptor antagonist
-65.00	BRD-K28346421	rifapentine	RNA polymerase inhibitor
-65.00	BRD-K36395411	SB-206553	Serotonin receptor antagonist
-65.01	BRD-K46862739	metyrapone	Cytochrome P450 inhibitor
-65.02	BRD-K52751261	TAK-715	p38 MAPK inhibitor
-65.17	BRD-A99833829	bethanechol	Acetylcholine receptor agonist
-65.37	BRD-K00610438	altanserin	Serotonin receptor antagonist
-65.43	BRD-K96527333	dehydroisoandosterone	GABA receptor modulator
-65.78	BRD-A07000685	hydrocortisone	Glucocorticoid receptor agonist

-65.88	BRD-K52620403	STO-609	Calmodulin antagonist
-65.93	BRD-K34437622	BRD-K34437622	Thymidylate synthase inhibitor
-66.02	BRD-K02283807	GR-32191	Thromboxane receptor antagonist
-66.25	BRD-A34208323	VU-0404997-2	Glutamate receptor modulator
-66.33	BRD-A15297126	fluocinonide	Glucocorticoid receptor agonist
-66.82	BRD-K64935403	ebelactone-b	Lipase inhibitor
-67.19	BRD-K67439147	SIB-1893	Glutamate receptor antagonist
-67.85	BRD-K91900765	VX-745	p38 MAPK inhibitor
-67.97	BRD-K78633253	EXO-1	ARF inhibitor
-68.06	BRD-A46186775	hydrocortisone	Glucocorticoid receptor agonist
-68.15	BRD-K46211610	tolazoline	Adrenergic receptor antagonist
-68.66	BRD-K68065987	MK-2206	AKT inhibitor
-68.98	BRD-A48720949	testosterone	androgen receptor agonist
-69.06	BRD-A61858259	CAY-10415	Insulin sensitizer
-69.78	BRD-A37492983	iocetamic-acid	Radiopaque medium
-70.18	BRD-K64052750	gefitinib	EGFR inhibitor
-70.31	BRD-K74763371	bosentan	Endothelin receptor antagonist
-70.40	BRD-K17674993	diflorasone	Corticosteroid agonist
-70.41	BRD-K14807180	SB-221284	Serotonin receptor antagonist
-70.61	BRD-A92670106	tocainide	Sodium channel blocker
-70.82	BRD-K11634954	GBR-13069	Dopamine uptake inhibitor
-70.93	BRD-A45498368	WYE-125132	MTOR inhibitor
-71.08	BRD-K63550407	erythromycin	NFkB pathway inhibitor
-71.11	BRD-K68507560	dicycloverine	Acetylcholine receptor antagonist
-71.25	BRD-K32318651	acyclovir	DNA polymerase inhibitor
-71.58	BRD-K39983086	loteprednol	Glucocorticoid receptor agonist
-71.63	BRD-A20126139	medrysone	Glucocorticoid receptor agonist
-72.69	BRD-K45435259	SCH-23390	Dopamine receptor antagonist

-72.78	BRD-K26674531	GR-235	Estrogen receptor agonist
-72.93	BRD-K97810537	beclometasone	Glucocorticoid receptor agonist
-73.53	BRD-K27184429	levocetirizine	Histamine receptor antagonist
-73.65	BRD-K10042277	desmethylclozapine	Acetylcholine receptor agonist
-73.98	BRD-K80725821	RS-16566	Serotonin receptor antagonist
-74.02	BRD-K93176058	AC-55649	Retinoid receptor agonist
-74.68	BRD-K19111024	clofibric-acid	PPAR receptor agonist
-74.73	BRD-K83637872	SANT-1	Smoothened receptor antagonist
-75.00	BRD-K17110974	aristolochic-acid	Phospholipase inhibitor
-75.15	BRD-A84702196	penicillin	Bacterial cell wall synthesis inhibitor
-75.32	BRD-A37776212	ICI-204448	Opioid receptor agonist
-75.48	BRD-K32164935	tolazamide	ATP channel blocker
-75.59	BRD-A92439610	triamcinolone	Glucocorticoid receptor agonist
-75.74	BRD-K06335600	tizanidine	Adrenergic receptor agonist
-76.53	BRD-A00520476	otenzepad	Acetylcholine receptor antagonist
-76.57	BRD-K66782112	BRD-K66782112	Histamine receptor antagonist
-76.71	BRD-K71035033	masitinib	KIT inhibitor
-76.71	BRD-A70407468	PSB-36	Adenosine receptor antagonist
-76.81	BRD-A41301928	bongkrek-acid	Mitochondrial ADP, ATP translocase inhibitor
-76.83	BRD-A55594068	vinblastine	Microtubule inhibitor
-77.03	BRD-K85603128	resorcinol	Phosphodiesterase inhibitor
-77.33	BRD-K62200014	anagrelide	Phosphodiesterase inhibitor
-77.62	BRD-K35240538	methylprednisolone	Glucocorticoid receptor agonist
-77.71	BRD-K15592317	CP466722	ATM kinase inhibitor
-77.78	BRD-K49668410	clarithromycin	Bacterial 50S ribosomal subunit inhibitor
-78.14	BRD-K64402243	ivachtin	Caspase inhibitor
-78.14	BRD-K33308633	INCA-6	Calcineurin inhibitor
-78.68	BRD-K97799481	theophylline	Adenosine receptor antagonist

-78.90	BRD-K88611939	aniracetam	Glutamate receptor agonist
-79.01	BRD-A75479906	rimantadine	Antiviral
-79.06	BRD-A18043272	phensuximide	Succinimide antiepileptic
-79.27	BRD-A41145729	methoprene-acid	Retinoid receptor agonist
-79.52	BRD-K60640630	mometasone	Glucocorticoid receptor agonist
-79.85	BRD-K77286328	reversine	Aurora kinase inhibitor
-79.96	BRD-K00337317	NU-7441	DNA dependent protein kinase inhibitor
-80.26	BRD-K20152659	gamma-homolinolenic-acid	Cholesterol inhibitor
-80.55	BRD-K56596464	QX-314	Sodium channel blocker
-80.65	BRD-A31801025	formestane	Aromatase inhibitor
-80.68	BRD-K25906698	immepip	Histamine receptor agonist
-80.70	BRD-K18059238	gamma-linolenic-acid	Anti-inflammatory omega-6 fatty acid
-80.72	BRD-A83326220	brazilin	Nitric oxide production inhibitor
-80.84	BRD-K13927029	retinol	Retinoid receptor ligand
-80.98	BRD-K85266041	DNQX	Glutamate receptor antagonist
-81.23	BRD-K79425933	benperidol	Dopamine receptor antagonist
-81.33	BRD-K02404261	caffeine	Adenosine receptor antagonist
-81.36	BRD-A91452556	estradiol-cypionate	Estrogen receptor agonist
-81.40	BRD-K17306061	aprepitant	Tachykinin antagonist
-81.42	BRD-K61323504	SB-225002	CC chemokine receptor antagonist
-81.59	BRD-K11742128	triprolidine	Histamine receptor antagonist
-81.83	BRD-A25067867	benzatropine	Acetylcholine receptor antagonist
-81.93	BRD-K14681867	somatostatin	Somatostatin receptor agonist
-82.03	BRD-A31800922	procyclidine	Acetylcholine receptor antagonist
-82.08	BRD-K75478907	GS-39783	GABA receptor modulator
-82.16	BRD-K19796430	erismodegib	Smoothened receptor antagonist
-82.28	BRD-K22828899	TUL-XXI039	Serine/threonine kinase inhibitor
-82.67	BRD-K36965586	m-chlorophenylbiguanide	Serotonin receptor agonist

-82.76	BRD-A45543382	metrizamide	Radiopaque medium
-82.83	BRD-A67605442	tetrahydrobiopterin	Nitric oxide stimulant
-82.99	BRD-A79903587	tegafur	Thymidylate synthase inhibitor
-83.03	BRD-K32645441	dipropyl-5ct	Serotonin receptor agonist
-83.18	BRD-K99063460	didanosine	Nucleoside reverse transcriptase inhibitor
-83.19	BRD-K94070024	depomedrol	Glucocorticoid receptor agonist
-83.35	BRD-A11813248	AM-92016	Potassium channel blocker
-83.46	BRD-K91733562	secoisolariciresinol	Antioxidant
-83.48	BRD-K28470988	L-690330	Inositol monophosphatase inhibitor
-83.90	BRD-K54095730	CMPD-1	p38 MAPK inhibitor
-84.10	BRD-A92630576	trimebutine	Opioid receptor agonist
-84.62	BRD-K60184833	tyrphostin-46	Tyrosine kinase inhibitor
-84.88	BRD-K64606589	apicidin	HDAC inhibitor
-85.26	BRD-K10974103	diloxanide	Protein synthesis inhibitor
-85.27	BRD-K37865504	LY-2183240	FAAH inhibitor
-85.45	BRD-K67844266	MLN-4924	Nedd activating enzyme inhibitor
-85.47	BRD-A02180903	betamethasone	Glucocorticoid receptor agonist
-85.88	BRD-K77987382	mebendazole	Tubulin inhibitor
-86.34	BRD-K62012036	acitretin	Retinoid receptor agonist
-86.38	BRD-A35108200	dexamethasone	Glucocorticoid receptor agonist
-86.80	BRD-K69328504	L-690488	Inositol monophosphatase inhibitor
-87.02	BRD-A35519318	benidipine	Calcium channel blocker
-87.29	BRD-A17448384	beclometasone	Glucocorticoid receptor agonist
-87.62	BRD-K64670467	JNJ-16259685	Glutamate receptor antagonist
-87.91	BRD-A29731977	17-hydroxyprogesterone-caproate	progesterone receptor agonist
-88.45	BRD-A29901043	KIN001-127	ITK inhibitor
-88.91	BRD-K53790871	triamcinolone	Glucocorticoid receptor agonist

-89.02	BRD-K31542390	mycophenolic-acid	Dehydrogenase inhibitor
-89.18	BRD-K31627533	rimexolone	Glucocorticoid receptor agonist
-89.74	BRD-K74913225	brinzolamide	Carbonic anhydrase inhibitor
-90.31	BRD-K20995441	U-54494A	Opioid receptor agonist
-90.40	BRD-K26548821	quinpirole	Dopamine receptor agonist
-90.52	BRD-K28667793	pyrazinamide	Fatty acid synthase inhibitor
-91.12	BRD-K27141178	SB-203186	Serotonin receptor antagonist
-91.37	BRD-K32330832	VER-155008	HSP inhibitor
-91.73	BRD-A65767837	hydrocortisone	Glucocorticoid receptor agonist
-92.20	BRD-K99498722	NPI-2358	Tubulin inhibitor
-92.25	BRD-K59184148	SB-216763	Glycogen synthase kinase inhibitor
-92.50	BRD-A23290232	westcort	Glucocorticoid receptor agonist
-92.75	BRD-K69837166	trap-101	Nociceptin/orphanin FQ (NOP) receptor antagonist
-92.94	BRD-A38749782	fludrocortisone	Glucocorticoid receptor agonist
-93.53	BRD-A28746609	paclitaxel	Tubulin inhibitor
-93.75	BRD-K49481516	galantamine	Acetylcholinesterase inhibitor
-94.06	BRD-A35033682	eriodictyol	Cytochrome P450 inhibitor
-94.06	BRD-A99571536	dubinidine	Anti-epileptic
-94.39	BRD-K09963420	saquinavir	HIV protease inhibitor
-94.77	BRD-K87990216	piretanide	Glucocorticoid receptor agonist
-94.85	BRD-A51714012	venlafaxine	Adrenergic inhibitor
-94.96	BRD-A68888262	azelastine	Histamine receptor antagonist
-95.02	BRD-K64881305	ispinesib	Kinesin-like spindle protein inhibitor
-95.38	BRD-K59273480	propentofylline	Adenosine reuptake inhibitor
-95.52	BRD-A56012032	thiorphan	Membrane metalloendopeptidase inhibitor
-95.56	BRD-A43150328	penicillic-acid	other antibiotic
-96.16	BRD-K17349619	HLI-373	MDM inhibitor
-96.25	BRD-A78295502	hydroquinine	Antiarrhythmic

-96.36	BRD-A31159102	fluoxetine	Selective serotonin reuptake inhibitor (SSRI)
-96.48	BRD-A42167015	carteolol	Adrenergic receptor antagonist
-96.94	BRD-A16700644	isoxsuprine	Adrenergic receptor agonist
-97.67	BRD-K38003476	clocortolone	Glucocorticoid receptor agonist
-97.72	BRD-K62206109	VUF-5681	Histamine receptor antagonist
-98.23	BRD-K23875128	RHO-kinase-inhibitor-III[rockout]	Rho associated kinase inhibitor
-98.24	BRD-A73741725	exemestane	Aromatase inhibitor
-98.59	BRD-K54210043	NS-1619	Calcium channel activator
-98.80	BRD-K72093121	vidarabine	Antiviral
-99.25	BRD-K40227168	vinburnine	Adrenergic receptor antagonist
-99.26	BRD-A01787639	naftopidil	Adrenergic receptor antagonist
-99.79	BRD-K76534306	enrofloxacin	Bacterial DNA gyrase inhibitor
